# IR Spectroscopy and Linear Support Vector Machine Analysis of Colorectal Liver Metastasis

**DOI:** 10.1021/acs.jpcb.5c07859

**Published:** 2026-02-02

**Authors:** James V. Coe, Heather C. Allen, Charles. L. Hitchcock, Edward W. Martin

**Affiliations:** † Department of Chemistry and Biochemistry, 2647 The Ohio State University, 100 West 18th Avenue, Columbus Ohio 43210-1173, United States; ‡ Department of Pathology, The Ohio State University, 4132 Graves Hall, 333 W. 10th Avenue, Columbus, Ohio 43210, United States; § Department of Surgery, Division of Surgical Oncology, The Ohio State University, 410 W 10th Avenue, Columbus, Ohio 43210, United States; ∥ 691053 IR Medtek LLC, 620 Taylor Station Road, Suite G, Gahanna, Ohio 43230, United States

## Abstract

The Colorectal Liver Metastasis (CLM) library contains 756,096 full range Fourier transform infrared (FTIR) microscope imaging spectra of 14 frozen tissue sections from 7 different consenting patients with liver metastasis of colorectal origin. A subset of 30 windows was defined in predominant tumor or nontumor regions for the training or testing of linear support vector machine (SVM) models. Since the number of consenting patients was small, the primary purpose of this work was to establish a physical chemistry perspective on the infrared (IR) spectroscopy of metastatic liver cancer using the large number of available spectra. The linear SVM model was trained with a Leave-One-Case-Out strategy to avoid case leakage, minimize overtraining, and offer simple feature selection. The primary result is the derivation and measurement of a spectral form for the average contribution to the tumor/nontumor decision at each spectral step, i.e., the “Decision Contribution Spectrum”. Finally, the results are used toward the design of a fast mid-IR cancer probe for the operating room giving quick tumor decisions despite a reduced range of wavenumbers and an increased interval between wavenumber steps as compared to the FTIR input spectral data.

## Graphical abstract

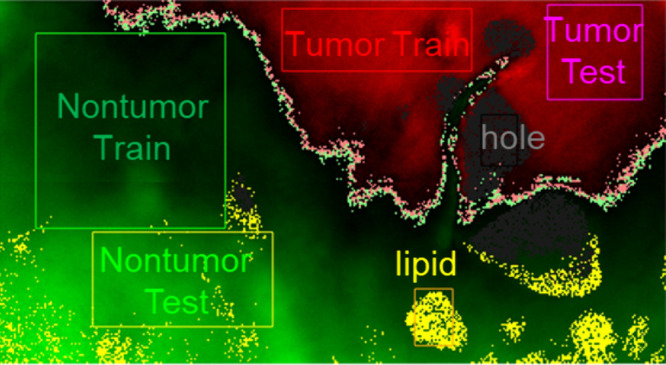

## 1 Introduction

This preclinical study produced the Colorectal Liver Metastasis (CLM) library which contains 756,096 full range infrared (IR) spectra, each corresponding to a spatial pixel size of 6.25 μm x 6.25 μm in a tissue section (spectral range 4000–750 cm^–1^ with 1626 steps of 2 cm^–1^ and spectral resolution of 4 cm^–1^). The CLM library has 1.23 billion pairs of information for analysis which have been applied to a variety of issues, but most importantly to identify the signatures of basic IR absorption spectroscopy for identifying liver tumors. Machine learning techniques are gaining wide attention for vibrational spectroscopy in biomedical applications.^1–5^ Mid-IR vibrational imaging spectroscopy is recognized for its ability to detect cancer^5–12^ without the need for labels such as fluorophores, radiolabels, and monoclonal antibodies.^5,13^ Ongoing work on diagnostic methods provides a growing foundation.^5,14–19^ The American Cancer Society tabulated 195,900 cases of rectal, colon, and colorectal cancer and 53,200 deaths in the year 2022 in the United States. Something like 70% of these cases metastasized to the liver. The liver is often the first place for metastasis because it regulates biochemical synthesis, degradation, and detoxification^20^ as the body’s chemical plant, interconnecting bile ducts, lymph nodes, and the blood circulatory system. The liver is unlike many other human internal organs in that it regenerates,^21,22^ so surgical resection of tumors is a particularly effective treatment relative to resections in other organs with metastatic tumors.

The immediate goal of this work is the derivation and measurement of the “Decision Contribution Spectrum” which quantifies the summed response of all support vectors at each wavelength to the cancer decision. Excellent signal-to-noise ratio was obtained because the CLM library is so large. The long-range goal of this work is to see if a fast mid-IR spectral probe based on a quantum cascade laser^23–28^ (QCL, with reduced spectral range relative to FTIR and increased interval between steps for speed) can aid surgeons in the removal or resection of metastatic liver cancers during live surgery by quickly revealing whether there is any cancer at the surgeon’s margin. Cancer diagnosis has long relied on the microscopic examination of cells to see if they have the appearance of cancer cells.^29–33^ We have recently shown that fast mid-IR spectral probe decisions match H&E stain results for keratinocytic carcinoma,^34^ so the prospects for a liver tumor probe have improved. Pathological assessment of a resected tumor specimen is labor intensive and time-consuming. Resected tissue (tissue containing tumor and a minimal buffer of nontumor at the surgeon’s margin) is frozen, sliced, fixed and stained (usually with an H&E stain, i.e., hematoxylin and eosin^29,30,33,35^ ), and the result is scanned under an optical microscope for diagnosis by cell morphology. Since the gold standard of cancer diagnosis is too slow to be of practical use during surgery and FTIR is too complicated (slow, moving optical parts, and liquid nitrogen cooled detectors), these results are being used to design a fast mid-IR spectral probe based on a quantum cascade laser^23–28^ (QCL) with reduced spectral range relative to FTIR and an increased spectral step size if not all of the full and continuous spectrum is needed. The probe would work to aid surgeons in the removal or resection of metastatic liver cancers during live surgery quickly revealing whether there is any cancer at the surgeon’s margin.

## 2 Linear Support Vector Machine Decision Equations for Spectroscopy

The FTIR spectra from each pixel in these imaging experiments can be used as a library of Predictors and the corresponding pathology assessments from H&E stains provide the Response variables for training a Support Vector Machine^36–40^ (SVM) decision equation, enabling the classification of new spectra as cancerous or not. SVM determines an optimized hypersurface for separating measurements of two classes (such as tumor and nontumor) using a selection of training data called “support vectors”, i.e., a constrained optimization of a separating surface using the data which lies between the two identified classes. The linear SVM method minimizes overtraining, provides feature selection, simplifies the matrix math, provides better transferability to new patients, and can be used independently of the generating software. The linear SVM equations are put in terms of IR spectra, and we have derived what we are calling the “Decision Contribution Spectrum”, i.e., the average contribution to the tumor/nontumor decision at each step along the spectrum (spectral steps). The linear SVM decision equation value from a reference text^40^ (p. 30 equation) can be re-expressed for a full IR spectrum to be tested, *Test*_*k,j*_, as

1 $$d_{k}=b+\underset{i}{\sum }\alpha_{i}y_{i}\left\langle \frac{SV_{i,j}-{\overset{®}{Train}}_{j}}{{\sigma_{Train}}_{j}}|\frac{Test_{k,j}-{\overset{®}{Train}}_{j}}{{\sigma_{Train}}_{j}}\right\rangle$$

where *i* is an index for support vectors, *j* for the spectral steps (and the dot product index), and *k* for the spectrum to be tested. The scalar offset or bias is *b*, *α*_*i*_ are the weights of the support vectors chosen to be near the separating hypersurface, and *y*_*i*_ is the class membership (nontumor or tumor in this case). It is essential to scale the test spectra, *Test*_*k,j*_ by subtracting the mean of the training set, ${\overset{®}{Train}}_{j}$ , and dividing by the standard deviation of the training set, σ_*Train*_*j*__. Mathematically, *d*_*k*_ is the perpendicular distance from the separating hyperplane to a test spectrum as a decision data point. Moving the support vector summation into the bracket, and commuting the row and column of the inner product gives^34^

2 $$d_{k}=b+\left\langle \frac{Test_{k,j}-{\overset{®}{Train}}_{j}}{{\sigma_{Train}}_{j}}|\beta_{j}\right\rangle ,,\mathrm{where}\;\beta_{j}=\underset{i}{\sum }\alpha_{i}y_{i}\left(\frac{SV_{i,j}-{\overset{®}{Train}}_{j}}{{\sigma_{Train}}_{j}}\right)$$

where *j* is the index over spectral steps. Constrained optimizations obtaining *b* and *β*_*j*_ were performed in the MATLAB programming environment (Matrix Laboratory by MathWorks) using the SVM linear kernel^35,36,40–42^ (MATLAB’s “fitcsvm.m” function). We call *β*_*j*_ the “SVM *β* spectrum”. The decision equation value, *d*_*k*_, has the form of distance, but there is a component for every step in the full IR spectrum (1626 components in this case), i.e., a hyperdimensional distance. Values of *d*_*k*_ > 0 classify into one class (tumor), and *d*_*k*_ ≤ 0 classify into the other (nontumor). Once obtained, the decision equation itself can be extracted (by outputting three spectra and a constant, i.e., ${\overset{®}{Train}}_{j}$ , σ_*Train*_*j*__, *β*_*j*_, and *b*) and used independently of MATLAB to classify new spectra. In our opinion, this machine learning formalism is closer to a least-squares fit than, for example, the filtering classifications of neural networks.

The primary utility of this approach becomes clear when considered in the context of the average spectrum of each class ( *T* _*j*_ for tumor and ${\overset{®}{NT}}_{j}$ for nontumor). Rather than summing the dot product contributions at each spectral step as with eq (2, first part), consider evaluating the average contributions at each spectral step of the average class spectra giving

3 $$d{\mathrm{T̅}}_{j}=b+\frac{{\mathrm{T̅}}_{j}-{\overset{®}{Train}}_{j}}{{\sigma_{Train}}_{j}}\cdot \beta_{j}\;\mathrm{and}\;d{\overset{®}{NT}}_{j}=b+\frac{{\overset{®}{NT}}_{j}-{\overset{®}{Train}}_{j}}{{\sigma_{Train}}_{j}}\cdot \beta_{j}$$

The difference is ${\overset{®}{\Delta d}}_{j}$ , the “Decision Contribution Spectrum”, i.e., the average contribution to the tumor/nontumor decision at each spectral step *j*

4 $${\overset{®}{\Delta d}}_{j}=d{\mathrm{T̅}}_{j}-d{\overset{®}{NT}}_{j}=\frac{\left({\mathrm{T̅}}_{j}-{\overset{®}{NT}}_{j}\right)-{\overset{®}{Train}}_{j}}{{\sigma_{Train}}_{j}}\cdot \beta_{j}$$

Notice that *β*_*j*_ alone is insufficient to indicate optimal spectral wavenumbers; i.e., it needs to be weighted by the scaled difference between average tumor and nontumor spectra. The large CLM library is used to make high signal-to-noise determinations of the “Decision Contribution Spectrum” constituting the most important result of this work. How the noise of the Decision Contribution Spectrum factors into decisions is a worthy topic of future investigation. Are the ${\overset{®}{\Delta d}}_{j}$ Decision Contribution Spectra different for different individuals or different cancers?

There is a second utility of linear SVM which we call “Decision Equation Imaging”. Histograms of the linear SVM decision equation values exhibit structured distributions; i.e., there are at least three different distributions (probably more) in the tumor region alone. In other words, there is information content in the magnitudes of linear decision equation values, above and beyond classification justifying the practice of decision equation imaging that is employed in this work. The decision equation images are created by scaling all positive decision equation values by the max and plotting in red with an RGB image for tumor. All negative decision equation values are scaled by the min (maximum in magnitude of the nontumor values) and plotted with green in an RGB image for nontumor. One might have to employ more statistical definitions for the max and min in general such as the mean plus or minus two standard deviations, particularly when the library is large and low probability observations are detectable. Decision equation imaging reveals interesting textures in the tissue groups which are different than, but able to be aligned with those revealed by H&E staining. There is information in the magnitude of linear decision equation values beyond a simple classification (*d*_*k*_ > 0 is tumor and *d*_*k*_ ≤ 0 is nontumor), for example it is possible to identify the tumor/nontumor transition with decision equation values near zero (for example, −0.5 to 0 to the nontumor side, 0 to +0.5 to the tumor side). Such imaging is employed throughout this work and can be viewed in case sample images of the Supporting Information.

## 3 Experimental and Methods

### 3.1 Experimental for Recording the CLM Library

The liver tissue^43,44^ slices or frozen sections studied in this work contained liver metastasis of colorectal origin which was surgically removed from 7 consenting patients (IRB # 2011C0085) yielding 14 tissue samples (Table S3 in the Supporting Information) during planned liver resections at the University Hospital, The Ohio State University, Columbus, OH. The primary purpose of this study was to establish a physical chemistry perspective on the IR spectroscopy using the huge library of IR spectra since the number of patients consenting was small. The tissue was snap frozen in liquid nitrogen and the sections were not fixed; i.e., there was no use of buffered formalin solution or dehydration with ethanol and xylene. Using the cryostat at −20 °C, frozen sections of ∼3 μm thickness were obtained to avoid saturating the strongest amide I band of protein in the IR spectra. Acquisition of data has been previously described.^43^ Briefly, as shown in Figure 1a, the sections came into the spectroscopy lab as o-ring sealed samples (see the PhD thesis of Zhaomin Chen,^35^ Figure 2.3) within a sandwich of 1 mm thick ZnSe windows, wedged at an angle of ∼5° to avoid etalon fringes, and housed in aluminum plates (1” x 3” matching typical glass microscope slides) with recessed through-holes holding the windows in place. While the PerkinElmer Spotlight 300 (see Figure 1b) came with an extended-time detector-cooling dewar, it did not work well, so we were limited to scanning regions that took about 3 h with the 16 element, liquid-nitrogen-cooled, MCT detector. Samples were never opened in the spectroscopy laboratories housing the IR imaging microscope. FTIR imaging spectra were recorded using a 16 element MCT array detector, 4 cm^–1^ resolution, 4000–750 cm^–1^ range, 16 scans per pixel, with a 6.25 μm square pixel edge. The names, sizes, and staining details of the frozen sections from 14 tissue samples are given in Table S3 of the Supporting Information. Bigger regions of multiple windows were stitched together outside of the commercial software using MATLAB (“fsmload.m” function by PerkinElmer). H&E stains were obtained for some cases by sending adjacent sections to a virtual slide facility [OSU Wexner Medical Center JML Molecular Laboratory @ Polaris, now moved onto OSU campus]. Other H&E stains were obtained by us immediately on the exact sample after the IR imaging and then imaged with an ordinary optical microscope. The yellow color cast of the ZnSe infrared optics was removed from the in-house optical microscope H&E stain images (Adobe Photoshop Elements 2.0), contrast-adjusted, scaled, rotated, and overlaid with the IR microscope images. H&E stains allowed team pathologists to point out definitive tumor and nontumor regions for use in training and testing. If one sums the areas of regions of all case tissue samples, it can be estimated that the IR imaging producing the CLM library took ∼43 h of scanning with the PerkinElmer Spotlight 300 IR microscope producing 756,096 full range infrared (IR) spectra each corresponding to pixel size of 6.25 μm × 6.25 μm and resolution of 4 cm^–1^.

**Figure 1:**
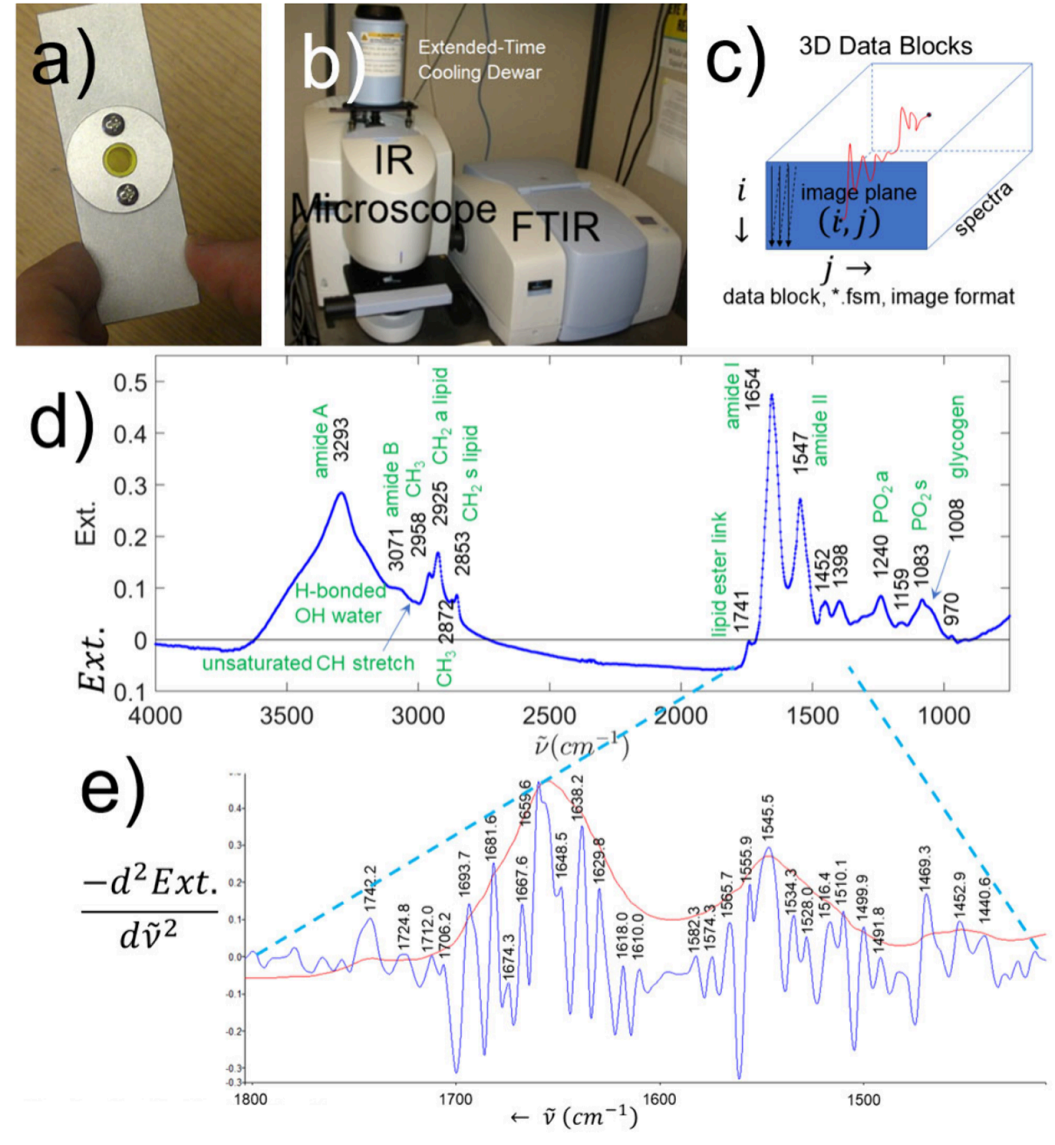
a) Microscope slide-sized frozen section tissue holder with wedged ZnSe windows. b) PerkinElmer Spotlight 300 for hyperspectral imaging of tissues set between the ZnSe windows of the slide in (a). c) Schematic form of the spectral data relative to the image plane as acquired with the commercial software of the Spotlight 300. d) Average IR raw spectrum of the whole CLM library with basic peak assignments. This is an extinction spectrum (Ext.) because it has both scattering and absorption components. e) Second derivative of extinction at the amide I and II bands. The 2nd derivative is a 5-point finite-difference approximation, and the derivative has been multiplied by −250 so that upgoing peaks give the centers of bands. The amide I and II bands are suppositions of many bands of different protein structures.

The average raw IR spectrum of all spectra in the CLM library without preconditioning (raw) is presented in Figure 1d. Protein gives rise to the most prominent peaks at 1656 cm^–1^ (amide I band) and 1547 cm^–1^ (amide II band) which are backbone vibrations shared by all proteins. Band shapes are very important in protein dominated spectra because the amide I and II bands are strong backbone vibrations and spectral measurements correspond to weighted averages of the various protein secondary structures (like α helix, beta sheets, turns, etc.). Lipids are evident at 1741, 2925, and 2853 cm^–1^. Many biomolecules contain phosphate groups which are evident at 1240 and 1083 cm^–1^, as well as the polysaccharides like glycogen as a shoulder at 1008 cm^–1^. Therefore, the subject tissues are a complex mixture of many biomolecules, and the amounts and identities of such biomolecules change when tissue transforms into cancer. To further develop this point, examine a 5-point, finite-difference approximation to the second derivative of the amide I and II bands (blue trace in Figure 1e) which has been multiplied by −250, so that upgoing peaks indicate the centers of unresolved bands. The absorption lineshapes (red trace of Figure 1e) of these bands have many inflections owing to different basic protein backbone structural motifs,^45,46^ like α helix, β sheet, loops, *etc.*, giving rise to shifts in the dominant amide I frequency. An illustration of these effects has been given for breast cancer metastatic to the liver^43^ (see Figures 5 and 6) using a method that simultaneously fits a line shape and its second derivative. So, changes in the protein of tissue associated with cancer will have different amounts of the basic protein structure types yielding differences in the inflections associated with the absorption line shape.

Machine learning models use spectra as the Predictor variables and a pathologist’s assessment as the Response variable. Several preconditioning options for analysis were explored including raw (no preconditioning), baseline-corrected (to remove nonzero scattering baselines), baseline-corrected and ratioed (to the amide I band response after baseline-correction), and baseline-corrected and normalized. First, progress in analysis was slow until a total of 30 windows were defined in predominant tumor or nontumor regions. Basically, the pathologist identifies the transition between tumor and nontumor, and windows were picked well away from the transition. The windows were all tumor or all nontumor simplifying the Response variables. Initial runs with a linear SVM model using 17% of the full library in training windows and 6.5% in testing windows obtained 0.00% training error and 0.30% testing error (using normalized and baseline corrected spectra). While this was a big improvement over our earlier efforts, there was ″case leakage″ in the training.^47^ This is also called “train-test contamination” or “improper splitting” in machine learning; i.e., spectra from each case were used in both training and testing. Machine learning models can be very good at picking-out individual specific features rather than cancer transformations. Since this work reveals individual differences, care was needed in train-test partitioning.

A Leave-One-Out (LOO) strategy^48,49^ was used for analysis to avoid case leakage. It consists of “... removing from the training data one element, constructing the decision rule on the basis of the remaining training data and then testing on the removed element”.^49^ This gave 7 different trainings avoiding case leakage which could be compared to each other and to a final training with all the data. The process of defining windows is shown for two representative case tissue samples in Figure 2 illustrating the nature of the hyperspectral imaging data. Space precludes showing all 14 case tissue samples, but they have all been made available in Supporting Information (Figures S1–S11). Figure 2a shows an IR microscope optical image of tissue on ZnSe as overlaid by the FTIR scanning region which is colored by decision equation values (green for nontumor, red for tumor). The red and magenta rectangles were for tumor windows, while green and yellow-green were for nontumor windows. To visualize the tumor/nontumor transitions, light green and light red were used for decision equation values near the transition boundary of the nontumor (−0.5 < *d*_*k*_ ≤ 0) and tumor (0 < *d*_*k*_ ≤ +0.5) sides of the transition, respectively. Other windows were also defined including the orange rectangle in (Figure 2a) for lipids. Figure 2b shows the H&E stain of the same tumor at similar scale, but not the same place in the tissue (tumor is to the right and nontumor to the left). In addition to tumor and nontumor windows, the lower tissue sample case (Figure 2c,d) also shows lymphocyte-rich regions (purple rectangular windows) where the immune system was fighting the cancer.

**Figure 2:**
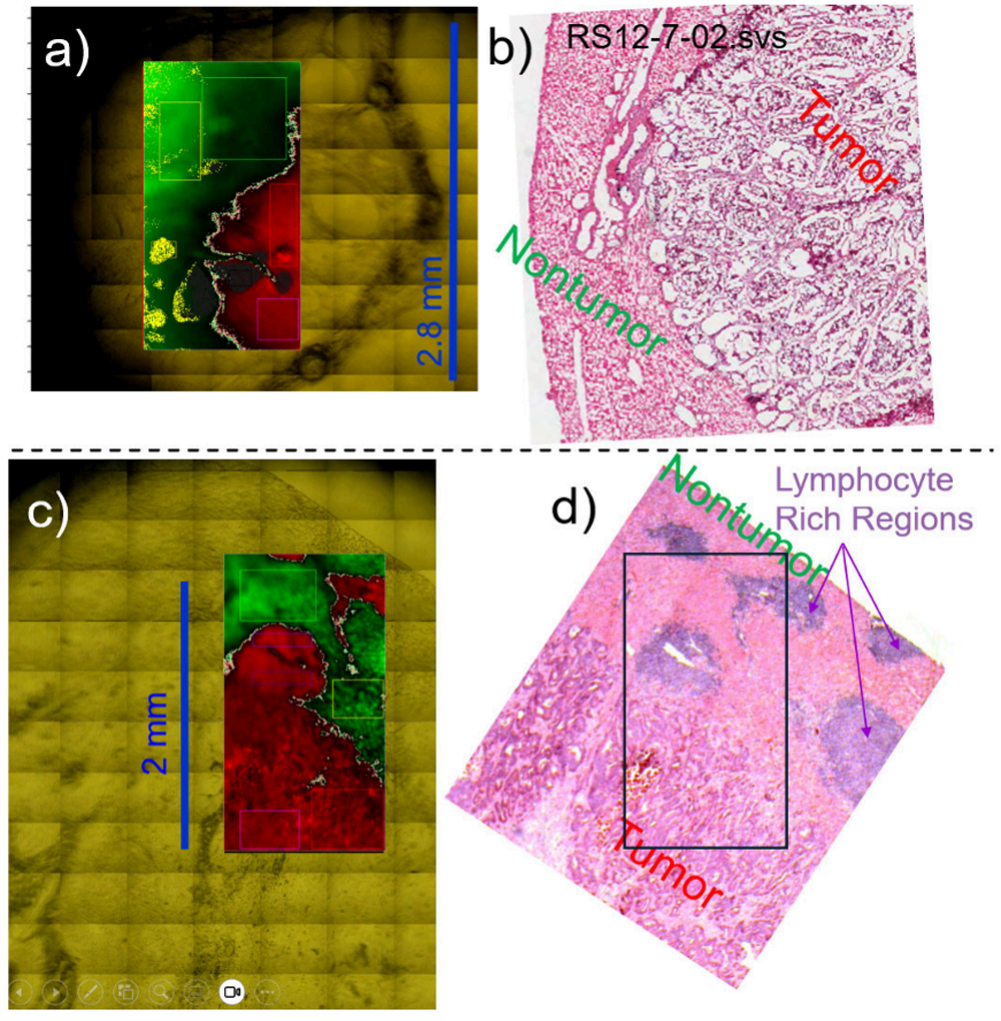
a) IR microscope optical image of Case 7 tissue on ZnSe as overlaid by the FTIR scanning region which is colored by decision equation values (green for nontumor, red for tumor). b) H&E stain of the same tumor of Case 7 at similar scale, but not the same place in the tissue. c) IR microscope optical image of Case 8 tissue on ZnSe as overlaid by the FTIR scanning region which is colored by decision equation values. d) H&E stain of the exact same tissue of Case 8 taken after IR scanning. In the decision equation images, the red and magenta rectangles are for all tumor, and the green and yellow-green rectangles are for all nontumor for use in training and testing strategies.

## 4 Results

The primary results to be presented here are for preconditioning with baseline-correction and normalization due to our interest in future probe applications. Notably, the raw data has scattering offsets which offer extra clues, but are not generally useful for spectral work beyond frozen sections. Baseline-correction increases the effectiveness of literature searches and provides better isolated absorption features, while normalization removes features depending on absolute properties like thickness of the section. The average tumor and nontumor training spectra of seven Leave-One-Case-Out trainings are shown at the bottom of Figure 3 exhibiting statistically valid differences between tumor and nontumor spectra. With all seven trainings displayed, one can see that the tumor/nontumor differences are bigger than the variations with training; i.e., there are repeatable differences. The primary results of this work are the seven Decision Contribution Spectra for colorectal cancer metastatic to the liver shown at the top of Figure 3 using the baseline-corrected and normalized option. Many features of the Decision Contribution Spectra are sharper than the absorption spectra themselves. The principle features repeat with each of the seven trainings. The largest magnitude deviations from zero are the most important contributors and they have been labeled with wavenumber positions at the top of Figure 3. Both the H-stretching (C–H, N–H, and O–H, 3600–2800 cm^–1^) region and the fingerprint region (2000–900 cm^–1^) are good for rendering decisions, although the fingerprint region appears to have more response (a combination of high and low peaks is desirable). An examination of Figure 3 top shows three regions (4000–3700 cm^–1^, 2700–2000 cm^–1^, and 900–750 cm^–1^) that make little contribution to the tumor/nontumor decision. The first two of these regions are devoid of fundamental vibrational bands. They are in no way surprising to spectroscopists and the result is predicted by Equation 4 [because ${\mathrm{T̅}}_{j}={\overset{®}{NT}}_{j}≅0$ ]. In signal-to-noise terms, no feature in the range from 2500 to 2100 cm^–1^ is bigger than 0.03 units whereas magnitudes elsewhere are as high as 1.4. However, the third range is full of biomolecular fundamental vibrations, but makes almost no contribution to the decision [due to a combination of ${\mathrm{T̅}}_{j}-{\overset{®}{NT}}_{j}$ being small and *β*_*j*_ ≅ 0]. Since regions contributing little to the decision still have noise, the Decision Contribution Spectra can reveal regions that need not be measured. The large number of spectra in the CLM library gives rise to excellent signal-to-noise in the Decision Contribution Spectra. This kind of simple feature selection is another advantage of the linear SVM model.

**Figure 3:**
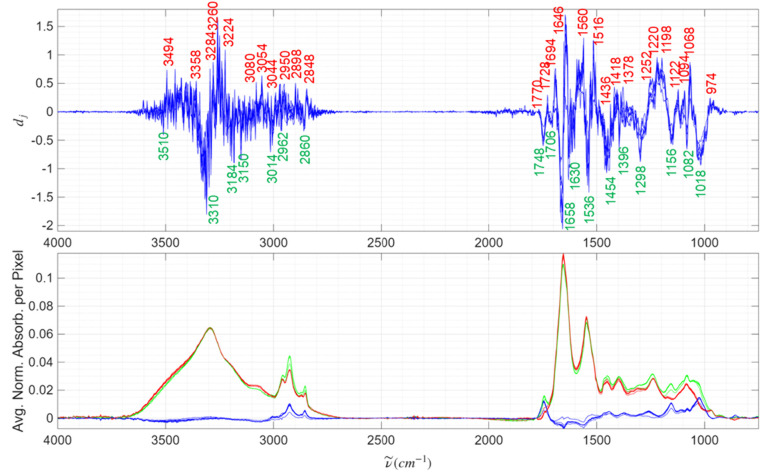
(Top) The Decision Contribution Spectra, ${\overset{®}{\Delta d}}_{j}$ , of the seven LOO trainings using baseline-corrected and normalized CLM spectra. Many maxima and minima have been labeled with the wavenumber positions. (Bottom) The average LOO training IR spectra of the tumor (red), nontumor (green), and difference (blue, Nontumor-Tumor). Gridding allows the reader to compare Decision Contribution Spectrum features to the average band centers. The Decision Contribution Spectra have many common features and there are systematic differences between tumor and nontumor IR spectra in all LOO trainings.

The decision equation files (three spectra and a constant, i.e., ${\overset{®}{Train}}_{j}$ , σ_*Train*_*j*__, *β*_*j*_, and *b*) are offered in the Supporting Information files and can be used to test spectra that are baseline-corrected, normalized, and of the same spectral format as this work.

Histograms of the linear SVM decision equation values for each of the LOO trainings and corresponding testing (with the left-out case) are shown in Figure 4. The LOO testing errors were 0.39%, 1.36%, 0.33%, 0.00%, 3.67%, 8.82%, and 0.00% for each case on the right-side of Figure 4 from top to bottom, which sums to 1498 errors out of 13,893 tests or 1.1% LOO testing error. While the largest case error is the next-to-last (case 10, 8.82%), the tumor and nontumor are still well separated in that histogram (see Figure 4) but the tumor group is distributed about *d* = 0. Based on the H&E images, we believe that this is due to a concentration of stroma cells^50,51^ (cancer-associated fibroblasts and/or mesenchymal stromal cells) in the tumor windows near the transition region. These stroma cells are neither normal liver cells, nor tumor cells. They can facilitate metastasis^52^ and are potential therapeutic targets. The testing on the right-side of Figure 4 reveals interesting individual variations, while the LOO training (left-side) reveals distributions of rough similarity with multiple features due to both individual differences and inhomogeneous tissues.

**Figure 4:**
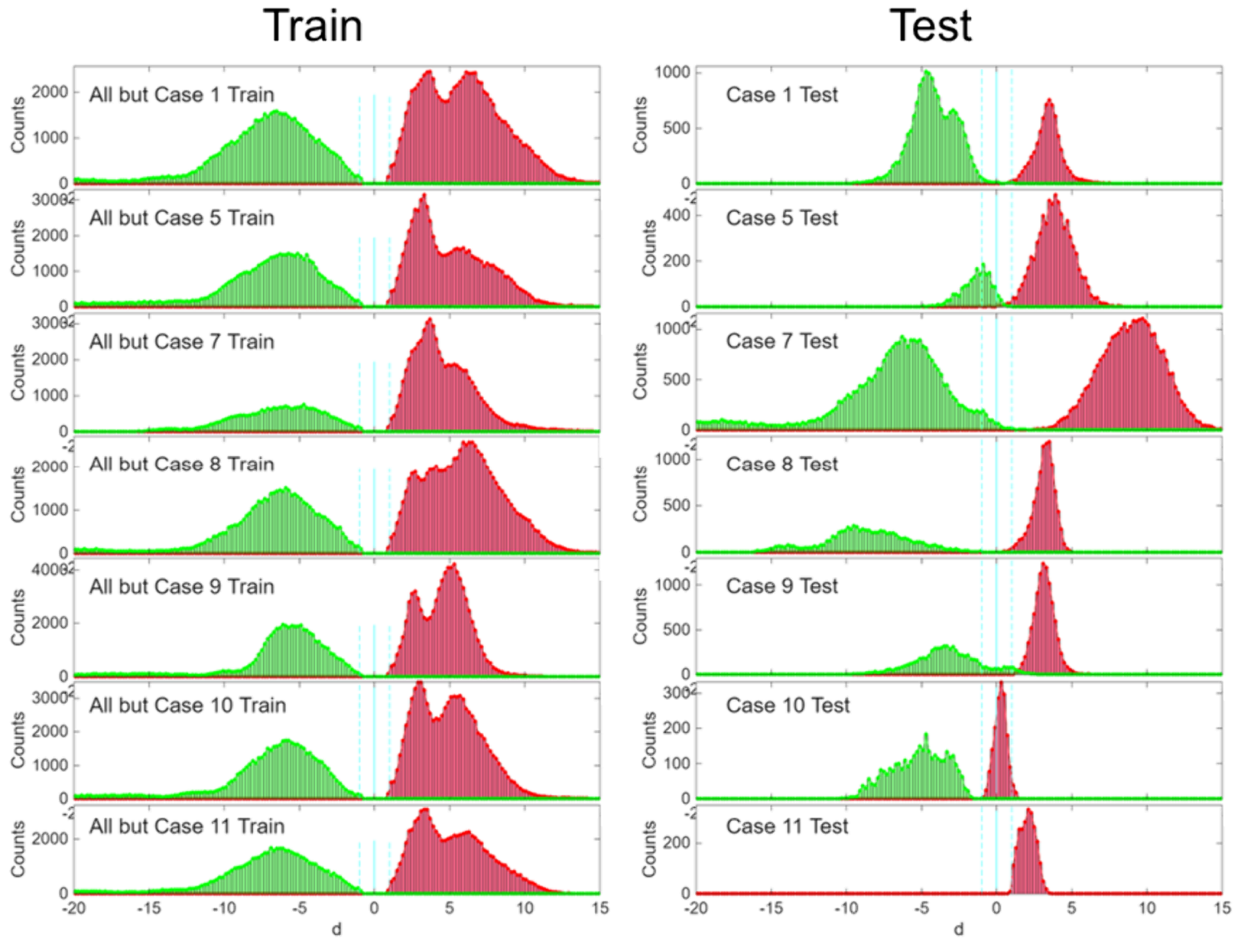
Histograms of decision equation values of the seven leave-one-out trainings. The testing of each case (right) is shown with the corresponding training on all the other cases (left). Green is for nontumor and red for tumor. The decision boundary is at *d* = 0 (solid cyan line), while the range of plus or minus one scaled standard deviation is shown with dotted cyan lines).

Lipids were identified by calculating the Pearson cross correlation of each spectrum in the CLM library with a reference lipid spectrum [Equation (s3) in the Supporting Information] as obtained by scaled subtraction of protein in different tissue spectra of the Case 1 red spot (inset of Figure 5a). Cross correlations can vary from −1 to +1 where a high value indicates similarity to the target molecule spectrum. Any pixel spectrum with a cross correlation greater than 0.5 was plotted with yellow as shown for four case tissue samples in Figure 5. Only 4 out of 14 tissue sample cases show high lipid, and the high lipid regions seem to gather at the tumor-nontumor transition. Considering that steatosis,^53–56^ or fatty liver disease, is an important condition unto itself and an important factor to consider in judging the risks of performing liver tumor resection; it is noteworthy that molecules with smaller contributions to the full IR spectrum can be isolated with high signal-to-noise using the CLM library.

**Figure 5:**
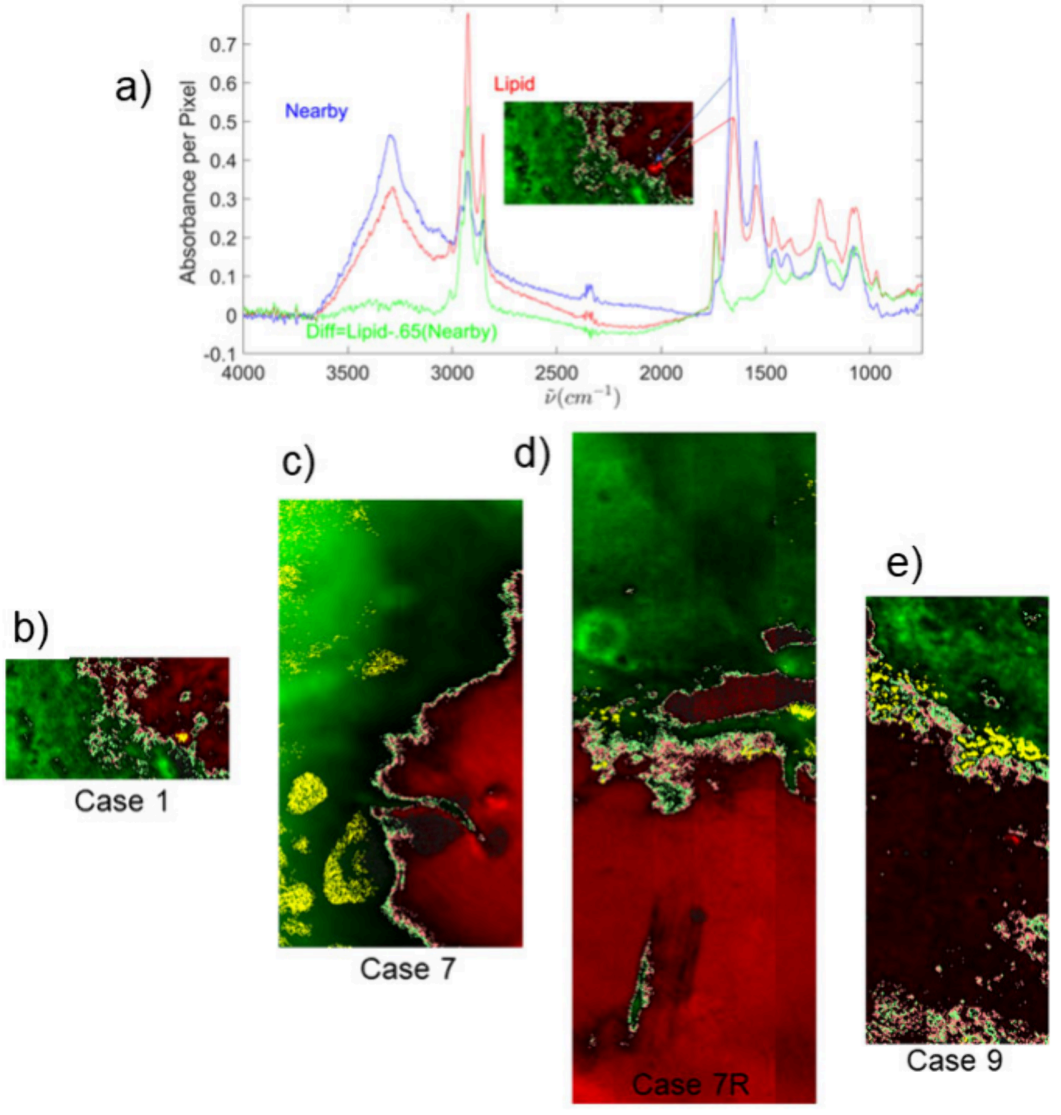
(a) The IR spectrum (red) of a pixel in the red spot of the decision equation image of Case 1 (inset) and the IR spectrum (blue) of a pixel positioned 5 pixels above the red trace. The scaled difference spectrum (green) reveals a lipid dominated spectrum. Pearson cross correlations greater than 0.5 with the lipid difference spectrum were plotted with yellow as shown for the case tissue samples with highest lipid (b–e).

Lymphocytes-rich regions^57^ were noted in the purple regions of H&E stains associated with Case 8 (Figure 2d and Figure 6b). Lymphocyte-rich regions reveal a battle ground against cancer. They are not yet tumor and are not counted as such by a Pathologist. However, they often become tumor if the patient loses this battle, so a surgical oncologist would want to remove these regions if encountered at the surgical margin. A decision equation training based on two windows (labeled “Xtest_Ls1” and “Xtrain_Ls1” in Figure 6a) was used to color an image using purple for lymphocyte decision equation values as shown in Figure 6c. This image has good correspondence with the H&E stain showing that lymphocyte-rich regions can be identified with mid-IR spectroscopy.

**Figure 6:**
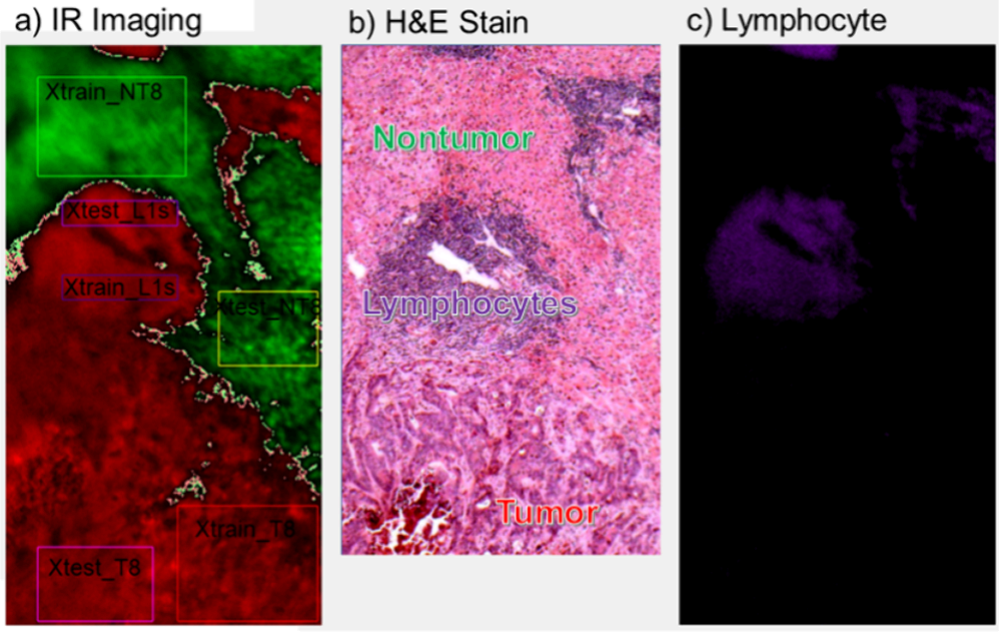
Case 8 images identifying lymphocyte-rich regions. a) IR tumor-nontumor decision equation image used for training and testing (light purple boxes, labeled “Xtest_L1s” and “Xtrain_L1s”). b) A higher-resolution H&E stain of Case 8 which is missing the bottom portion. c) Image of the lymphocyte rich region with the lymphocyte decision equation using purple while all the rest is left black.

Given reliable training, a decision equation value and a classification can be obtained at every pixel in the full CLM library (756,096 full-range IR spectra). Since many of our initial efforts started with the unsupervised method of K-Means Clustering (k-means) analysis, a 25 class k-means analysis was performed and is described in Supporting Information (Figure S17). Table S8 is given there which offers the average decision equation value for each cluster and the corresponding standard deviation. We originally suspected that most clusters would be dominated by tumor or nontumor; however, most clusters have both tumor and nontumor components. There were only three out of 25 that were dominated by one class. A principal component analysis (PCA) on the full CLM library was also performed as described in Supporting Information (Figure S19 has spectral plots of the principal components). It can be very interesting to project a third dimension of the decision equation values from a plot of one PC score against another (Figure S20). Unsupervised studies such as these can be further enhanced and more insightfully interpreted in terms of the linear SVM classifications, adding utility beyond our use in identifying tumor/nontumor transitions.

## 5 Toward Design of a Probe

FTIR is a mature and powerful spectroscopic technique, but it has optical moving parts, liquid nitrogen-cooled detectors, and it interferometrically measures all the wavelengths at the same time; so, it is basically too slow in its current commercial form for use in the operating room or for frozen section imaging.^58^ There are newer and competitive methods, including the broadband tunable quantum cascade laser (QCL),^58^ that can measure selected wavelengths at higher resolution without the need to sample all wavelengths. Such strategies are called discrete frequency IR^58–61^ by Bhargava and co-workers. So, is the full-range IR spectrum required to identify liver cancer or are some regions more important than others? Which of the fundamental band regions^5,46^ is better: the H-stretching region (Region 1 from 3670 to 2790 cm^–1^) or fingerprint region (Region 2, 1860–760 cm^–1^)? Can a single QCL range work in a spectral device for metastatic liver cancer?

The exercise started by training with 30 all-tumor or all-nontumor windows using full-range FTIR spectra and giving 0.00% training error and 0.30% testing error (albeit with case leakage). Then, linear SVM modeling was redone on Region 1 and Region 2 discarding all data outside of the region. Region 1 (H-stretch) got 3.12% wrong in training and 7.29% wrong in testing, while Region 2 (fingerprint region) got 0.04% wrong in training and 0.38% wrong in testing. Similarly, other studies on other tissues have found the fingerprint region is best,^62^ although both regions work^59^ with “the fingerprint region-based classifiers consistently emerging as more accurate.” QCLs are available in the fingerprint region (Region 2), but not currently in Region 1.

Further reduction in spectral range is possible. QCLs are often combined to operate like one broadly scanning system [Daylight Solutions, Block Engineering, and formerly Pranalytica, as employed in QCL-based spectral IR microscopes^63^], so calculations were performed on four popular fingerprint subregions: 1850–1644 cm^–1^, 1642–1352 cm^–1^, 1350–986 cm^–1^, and 984–780 cm^–1^. Training and testing errors as pairs were (2.83%, 10.72%), (2.75%,7.61%), (0.77%, 1.83%), and (8.72%, 10.25%), respectively. So, the single QCL in the third fingerprint subregion, 1350–986 cm^–1^, works best and might be useable for a fast mid-IR surgical probe. The best fingerprint subregion was further investigated regarding whether all spectral steps were needed. Training and testing errors in pairs were using all steps (0.77%, 1.83%), using half the steps (0.86%, 1.87%), using 1 in 5 steps (1.30%, 2.23%), and using 1 in 10 steps (1.97%, 2.66%), which corresponds to using 2, 4, 10, and 20 cm^–1^ intervals, respectively. The last two intervals exceed both the line width of the QCL and the FTIR resolution contributing to more rapid measurements. Considering that less expensive tunable QCLs have a range of ∼200 cm^–1^, there is a good prospect of designing a probe using a single, tunable QCL source.^28^ Linear SVM training and testing were performed (baseline-corrected and normalized option) of the CLM library using 200 cm^–1^ windows which were scanned through the full spectral range as shown in Figure 7. The best results were obtained with 200 cm^–1^ ranges centered at 1254, 1154, 1054, and 954 cm^–1^ with an average training error of 2.2% and testing of 3.5%. Good results were also obtained with ranges centered at 1754, 1654, 1554, and 1454 cm^–1^ with average training error of 3.2% and testing error of 10.5%. The range centered at 2954 cm^–1^ is not as good, but perhaps workable, with a training error of 9.1% and testing error of 14.0%. Things get much worse elsewhere. Note also that single QCLs have been produced that tune over a 400 cm^–1^ range, so lower error rates are possible with larger ranges.

**Figure 7:**
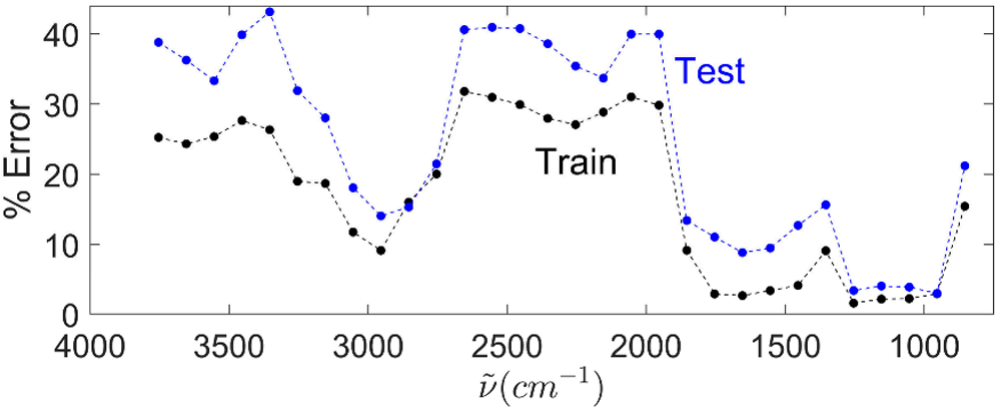
Training (black) and testing (blue) errors for the baseline-corrected and normalized option of the CLM library vs center of a 200 cm^–1^ range.

## 6 Discussion and Conclusions

The CLM library is large with 756,096 full range infrared (IR) spectra. Each spectrum corresponds to a 6.25 μm × 6.25 μm image pixel (in one of the 14 tissue frozen section samples obtained from seven consenting patients) which is smaller than the typical hepatocyte cell. The CLM data has been primarily applied to identify the signatures of basic IR absorption spectroscopy for identifying liver tumors. While our early efforts involved good results at the expense of case leakage (spectra from a case in both training and testing), a leave-one-out approach was employed to get results without the bias of case leakage and therefore a better estimate of generalization of the classifier. The leave-one-case-out analysis (linear SVM model and the baseline-corrected and normalized option) obtained 1.1% testing error with 13,893 tests. The testing error with case leakage was only 0.294%, so case leakage matters, but we are still getting good results. Histograms of the decision equation values of the seven different LOO trainings and the corresponding testing of the left-out case are given in Figure 4. There is evidence of both individual patient variations and inhomogeneous tissue contributions to the observed distributions (multiple overlapped distributions). The high accuracy of all these determinations is attributed to the use of training and testing subregions clearly inside the regions identified by the Pathologist and well away from transitions between tumor and nontumor. Linear SVM decision equation imaging gave excellent working images of the frozen sections identifying the transition between tumor and nontumor (−0.5 < *d*_*k*_ < +0.5) as was used in the images of all 14 tissue samples (see Figure 2a,c and Supporting Information). The decision equation output is made available as Supporting Information in the form of an Excel file, i.e., by outputting three spectra and a constant, namely, ${\overset{®}{Train}}_{j}$ , σ_*Train*_*j*__, *β*_*j*_, and *b* from Equation 1, as well as average tumor and nontumor spectra.

Most importantly, a new method of feature selection which we call the “Decision Contribution Spectrum” or ${\overset{®}{\Delta d}}_{j}$ [Equation 4] was derived and extracted from the data as shown at the top of Figure 3 with very high signal-to-noise ratio. It is the average contribution to the tumor/nontumor decision at each spectral step. The wavenumbers making the largest contributions have been labeled at the top of Figure 3, which has the seven LOO training results overlaid. As an example of the utility of the Decision Equation Spectrum, consider the amide I band region (1600–1700 cm^–1^). It has about ten discernible peaks due to different secondary protein structures (Figure 1e), while the Decision Contribution Spectrum (Figure 3 top) shows two-upgoing peaks at 1694 and 1646 cm^–1^ and two down-going peaks at 1658 and 1630 cm^–1^ in the amide I band range. These correspond to sub-bands of different protein secondary structures, and they reveal the places in the amide I band shape that have changes most related to cancer transformation. Consequently, band shape information is not lost with the linear SVM method using full-range spectra; the method picks out subtle changes in protein inflections^43^ that are different in tumor and normal tissue.

Decision equations determined in this work provide a high accuracy determination of every pixel in the CLM library, which in turn can be used to perform analyses not previously possible using the full 756,096 spectra of the CLM library. Standard unsupervised methods of k-means clustering and principal component analysis (PCA) lend themselves to new and more powerful interpretations given good decision equation values. In the early days of analyzing this data set, we would run k-means clustering and try to identify clusters of spectra that were predominantly in the tumor (or nontumor). The SVM decision equation values show that only a few of the clusters are predominantly tumor (or nontumor). Most k-means clusters have both tumor and nontumor features, so it was not until we employed reduced subregions of tumor and nontumor that high accuracy was obtained in training. Also, we explored a reduction of the dimensionality of the full CLM library (not just testing or training parts) by representing it with a subset of 15 principal components and their corresponding scores with an accuracy of ∼94% which is not as good as the LOO trainings but might be useful in other applications. It is also worth noting that our early efforts on metastatic liver cancer frozen section spectra used peak intensity ratio metrics as learned from Bhargava and co-workers^12^ on a different cancer type. Peak ratios are useful because they are independent of tissue slice thickness, and they offer a route to faster measurements by only measuring response at selected wavenumbers. Evaluations of sets of peak ratios have been provided in the Supporting Information. It might be possible to select good peak ratios using the Decision Contribution Spectrum or simply to select a good subset of wavenumbers for measurement using the Decision Contribution Spectrum.

Our ultimate goal was to design a fast mid-IR cancer probe for use during liver resections. By using a quantum cascade laser (QCL), one can choose a subset of wavenumbers for measurement rather than the full spectrum afforded by FTIR methods, in order to get faster results. The H-stretching region (3670–2790 cm^–1^) is good (3.12% wrong in training and 7.29% wrong in testing), but not as good as the fingerprint region (1860–760 cm^–1^, 0.04% wrong in training and 0.38% wrong in testing) for distinguishing colorectal liver metastasis which is similar to another study on different tissue.^62^ Calculations were also performed on four popular QCL fingerprint subregions and the region from 1350 to 986 cm^–1^ was best with training error of 0.77% and a testing error of 1.83%. A single QCL in the third fingerprint subregion could also skip spectral steps. Using 1 in 5 steps (10 cm^–1^ intervals) the training and testing errors were 1.30% and 2.23%, respectively. This step exceeds both the line width of the QCL and the FTIR resolution enabling more rapid measurement. Furthermore, a reduced range and increased step size option (54 wavenumbers from 1340 to 1870 cm^–1^ in steps of 10 cm^–1^) was explored which is being used in clinical trials for skin cancer.^34^ The SVM training accuracy was 96.4% which is very good considering that only 54 out of 1626 wavenumbers were used in a range of only 530 cm^–1^ (out of 3250 cm^–1^ of the full scale). Note that even though we are stepping in 10 cm^–1^ intervals, the resolution of the measurements is still 4 cm^–1^ with this approach (and <1 cm^–1^ with a QCL). Commercial QCLs are just now becoming available with ranges of this size. It is clearly possible to create fast mid-IR probes that can detect tumors with >95% accuracy on times scales of ∼10 s which might be employed by surgical oncologists during liver resections. Such probes may be used to measure whether the surgical oncologist has gotten all the tumor out, i.e., that there is no tumor at the surgical margin.

The CLM library offers research and utility in the fight against cancer. A small number of consenting patients leads to unreliable and less generalizable effects, so the results of this work are not yet useful for medical decisions. The primary purpose of this work was to establish a physical chemistry perspective on the infrared (IR) spectroscopy of metastatic liver cancer using the large number of available spectra. The application of these results to reduced ranges and increased step intervals suggests that there is potential utility in a fast IR probe for use in liver tumor resections, but much more work and study would be needed. The CLM library can be used to do more research on colorectal cancer metastatic to liver, to identify minority component biomolecules such as lipids, to analyze the protein line shape details of regions with different protein composition, to discriminate against different cell types like lymphocytes, and to identify holes (arterioles, venules, bile ducts, or tears).

## Supplementary Material





## Data Availability

The CLM library is available as MATLAB programs upon request to the corresponding author and approval by the lead investigators (Coe, Allen, Hitchcock) and their institutions.

## Notes

IR and H&E images of each of the 14 case tissue samples, details on training and testing window regions of the CLM library, instructions for loading the raw library with file names and sizes, and a few other full library results (PDF)
Decision equation data and average tumor and nontumor spectra for the seven LOO trainings (XLSX)

## #

P3 Infrastructure, Inc., 671 McCauley Rd., Stow, OH, 44224-1001, United States

## $

Senior Lab Technician, Air Company, 42-29 158th St., Flushing, NY, 11358, United States

## %

IR Medtek LLC, 620 Taylor Station Road, Suite G, Gahanna, OH 43230, United States

## ^

Department of Environmental Science, College of Environmental Science and Engineering, Tongji University, 1239 Siping Rd, Shanghai 200092, China

## △

Sanofi Institute for Biomedical Research, Beijing, China

## a

Heather C. Allen, allen@chemistry.ohio-state.edu

## b

Samantha A. Skarda, skarda.7@buckeyemail.osu.edu

## c

Trina D. Orr, orr.326@buckeyemail.osu.edu

## d

Antrae L. Wilson II, wilson.3989@buckeyemail.osu.edu

## e

Destiny G. Wilson, wilson.4148@buckeyemail.osu.edu

## f

Susan Kalliantas, kalliantas.4@buckeyemail.osu.edu

## g

Steven V. Nystrom, snystrom11@gmail.com

## h

Rebecca C. Bradley, bbradley@irmedtek.com, rebeccab1992@msn.com

## i

Ran Li, franceslee626@gmail.com

## j

Zhaomin Chen, zhaomin_chen@hotmail.com

## k

Charles. L. Hitchcock, emerituschuck@gmail.com

## l

Edward W. Martin Jr. Edward.Martin@osumc.edu
