# Supplementary material for: IR Spectroscopy and Linear Support Vector Machine Analysis of Colorectal Liver Metastasis
Source: J Phys Chem B. 2026 Feb 2;130(6):1798–809. doi: 10.1021/acs.jpcb.5c07859 (PMC12908113; doi:10.1021/acs.jpcb.5c07859)
Supplement: Supplementary file 1 [file jp5c07859_si_001.pdf]

Supporting Information for "IR Spectroscopy and Linear Support Vector Machine Analysis of Colorectal Liver Metastasis"

James V. Coe[a], Heather C. Allen[a], Samantha A. Skarda[a], Trina D. Orr[a], Antrae L. Wilson II[a], Destiny G. Wilson[a], Susan Kalliantas[a], Steven V. Nystrom[a], Rebecca C. Bradley[a], Ran Li[a], Zhaomin Chen[a], Charles. L. Hitchcock[b], and Edward W. Martin Jr.[c]*

[a]The Ohio State University Department of Chemistry and Biochemistry, 100 West 18th Avenue, Columbus OH 43210-1173, USA

[b]The Ohio State University, Department of Pathology, 4132 Graves Hall, 333 W. 10th Ave, Columbus, OH 43210, USA

[c]The Ohio State University, Department of Surgery, Division of Surgical Oncology, 410 W 10th Avenue, Columbus, OH 43210

[d]IR Medtek LLC, 620 Taylor Station Road, Suite G, Gahanna, OH 43230

CONTENTS

S1 Tissue Samples by Case 2
S1.1 ***Case 1*** 2
S1.2 ***Case 5*** *3*
S1.3 ***Case 7*** *4*
S1.4 ***Case 8*** *6*
S1.5 ***Case 9*** *7*
S1.6 ***Case 10*** *8*
S1.7 ***Case 11*** *8*
S2 Training and Testing Windows 10
S3 Full Library Loading 12
S4 Spectral Preconditioning, Holes, and Lipids 14
S5 Extra Results 17
S5.1 ***Decision Equation Output*** 17
S5.2 ***Peak Ratio Metrics of Previous Work*** 18
S5.3 ***Tumor/Nontumor Transitions*** 20
S5.4 ***K-Means Clustering with Decision Equation Values*** 21
S5.5 ***Principal Components with Decision Equation Values*** 23
S5.6 ***Lymphocytes*** 25
References 25

S1 Tissue Samples by Case. The best way to train data was not known in advance, so procedures and data collection regions changed throughout the study. The important task of assigning the training and testing regions is described for each case tissue sample, including the IR imaging and H&E stains that were used to define the training and testing regions. When a pathologist reads the H&E image and points out a tumor, it is not practical to specify every pixel in the image as tumor or nontumor. Therefore training and testing regions were chosen away from tumor-nontumor transitions avoiding the details of where the tumor transition was drawn. As this is the most crucial task in this preclinical study, we go through the images and details of each case sample.

S1.1 ***Case 1.*** The first case had three sample regions which we have labelled as Case 1E, Case 1, and Case 1G. When an investigator is making frozen sections, it is possible to see the tumor

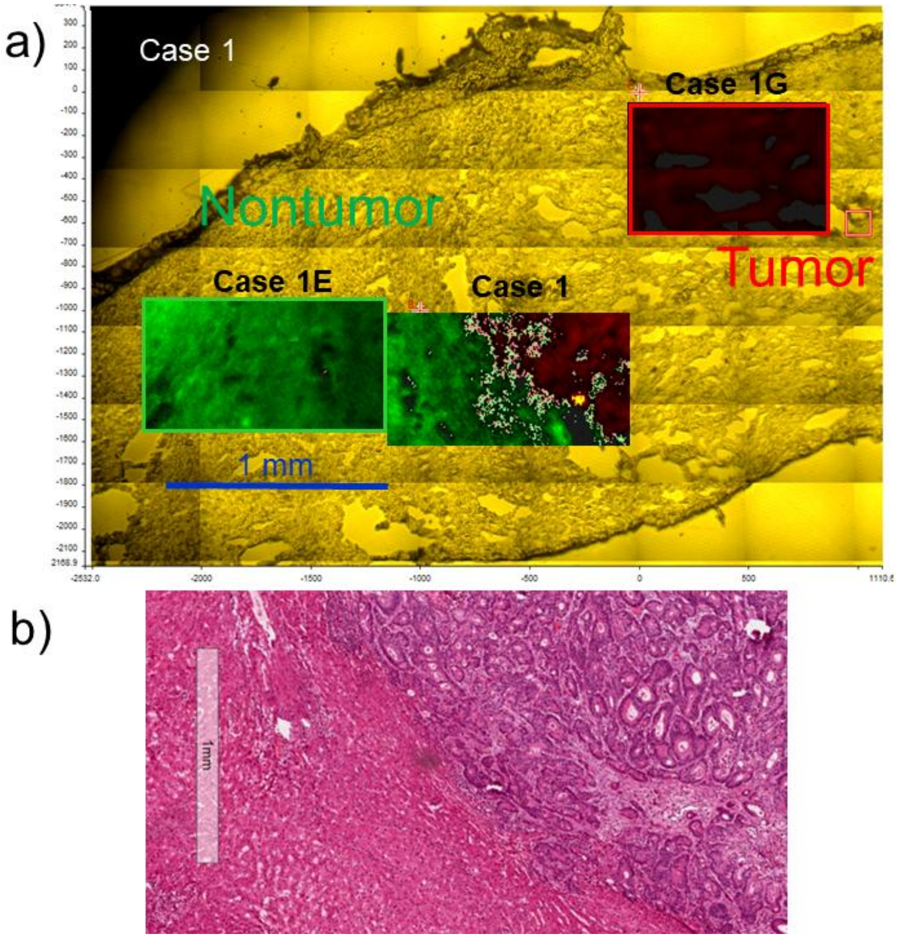

Figure S1. a) IR microscope image of scanning regions showing the three sampled regions called Case 1E (all nontumor), Case 1 (transition), and Case 1G (all tumor). All of Case 1E was used to train for nontumor, while all of Case 1G was used to train for tumor. Dark gray was used to image holes in the tissue and yellow-orange for lipid-rich regions. The decision equation range of -0.1 to 0 is the nontumor side of the boundary (light green), while 0 to +0.1 is the tumor side of the boundary (light red). b) H&E stain snapshot at 2x of the same tumor at a similar scale, but not at the same position (Aperio ImageScope, RS12-001-2.svs), i.e. to characterize the kinds of tissue being investigated.

which is usually lighter than normal liver tissue. However, after sectioning into a 3 μm thick slice, it is no longer possible to see the tumor. Case 1E was chosen to be within the nontumor region, Case 1 was (hopefully) at the transition, and Case 1G was chosen to be well within the tumor. The IR imager's microscope picture is shown in Figure S1a having a yellow color cast due to the ZnSe windows of an IR sample cell. The image is overlayed with three IR scanning regions (Case 1E and Case 1 are each 0.600 mm vertical by 1.100 mm horizontal which is 96 pixels x 176 pixels, while Case 1G is 0.600 mm vertical by 0.900 mm horizontal or 96 pixels x 144 pixels). These images use decision equation values with red for tumor and green for nontumor as will be explained in later sections. We do not have an exact overlay with an H&E image, but Figure S1b shows a snapshot from a virtual slide (Aperio ImageScope, RS12-001-2.svs, at 2x, 2/22/2012) of the same tumor and scaled to a similar size as the Figure S1a. In this case, all of Case 1E was used as part of the nontumor training (green box), and all of Case 1G was used as part of the training for tumor (red box). H&E-stained sections represent the gold standard of cancer diagnosis[1-4]. Note that the tumor is in the upper right of Figure S1b and has larger cells with a dark blue and white texture. The nontumor is on the lower left of Figure S1b in a region dominated by hepatocytes cells which stain with the pink-orange color of the eosin stain.

S1.2 ***Case 5.*** A different H&E strategy was employed with the Case 5 set, namely we performed an H&E stain on the exact same tissue that had been IR-imaged as shown in Figure S2. First, sample regions called Case 5 and Case 5A were scanned believing that the transition

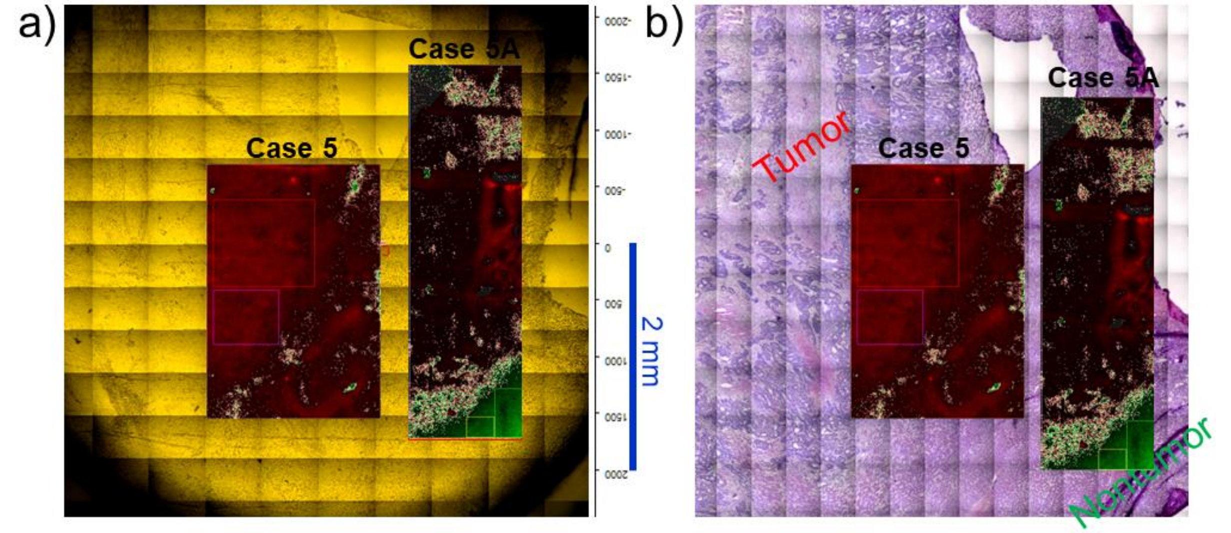

Figure S2. Cases 5 and 5A were recorded at adjacent sites. a) IR microscope image of the scanning regions with overlays of the decision equation images. Training and testing regions for tumor are given in Case 5 with red and magenta rectangles, respectively. Training and testing for nontumor are given in Case 5A with green and yellow rectangles, respectively (bottom right of Case 5A). b) H&E stain of the exact same tissue.

between tumor and nontumor was somewhere in between, however - in the end - only a small portion of the lower right-hand side of Case 5A was fully nontumor.

A second set of imaging experiments were done on Case 5C and Case 5CH as shown in Figure S3. Unlike Figure S2, the H&E slide in Figure8b is not the exact same piece of tissue as in Figure S3a, rather it shows a similar region with a slim belt of nontumor on the left, tumor on the right, and a substantial region between with both tumor and nontumor. A training (green) and

testing (yellow) rectangle were assigned for nontumor at the far-left side of Case 5CH. There was nontumor in Case 5C, but it was mixed with tumor and not used for training.

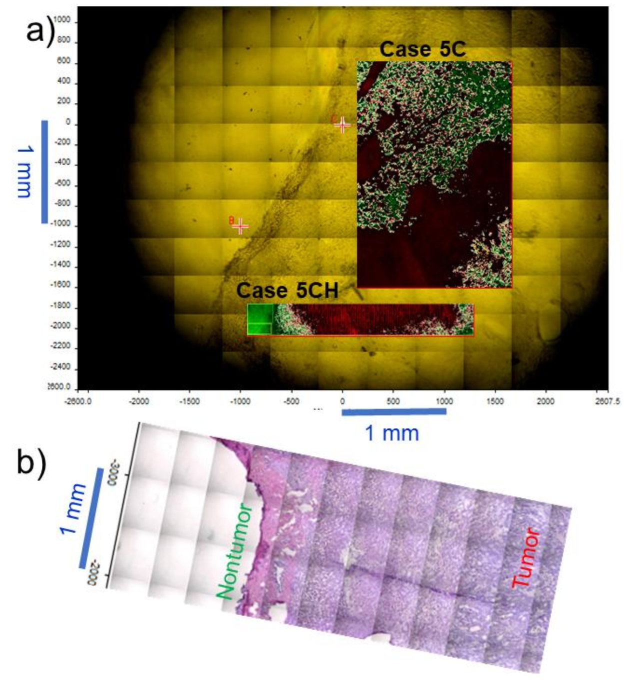

Figure S3. Cases 5C and 5CH were recorded at adjacent sites. Nontumor training (green) and testing (yellow) regions were defined at the left side of Case 5CH. a) IR microscope image of the scanning regions with overlays of the decision equation images. b) H&E stain of the same tumor, but not the same region.

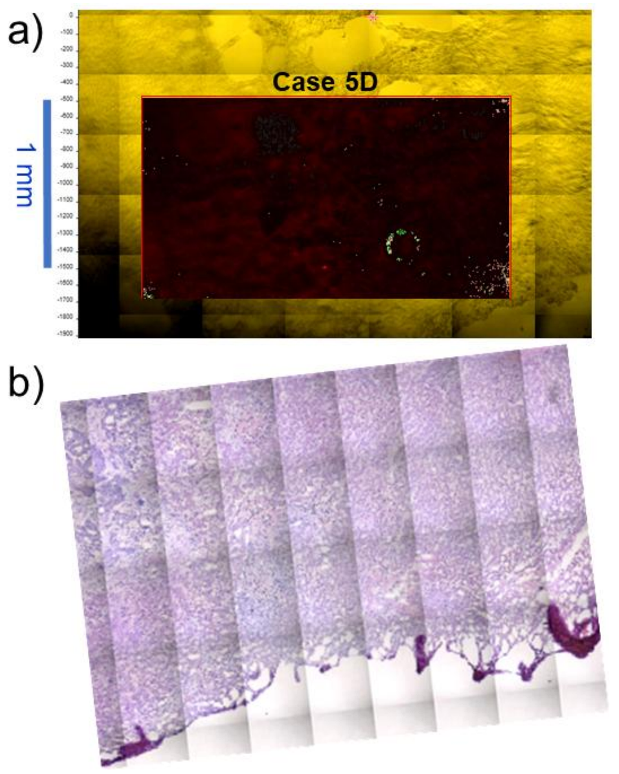

Figure S4. Case 5D turned out to be all tumor. a) IR microscope image of the scanning region with an overlay of the decision equation image (all red for tumor). This tissue was also torn when mounted on the IR sample holder, so the dark gray portions in the decision equation image overlay are holes. b) H&E stain of similar region of the same tumor, but not the same place in the tissue.

Finally, one more data set on the Case 5 tumor was recorded that is called sample case 5D as shown in Figure S4. It turned-out to be all tumor. The Case 5 sample images illustrate that in cancer studies, it is often more difficult to get good nontumor samples than it is to get tumor samples.

S1.3 *Case 7.* Two regions were sampled for the Case 7 tumor which was a metastatic neuroendocrine carcinoid type cancer yielding two sample regions called Sample Case 7 and Sample Case 7R. Sample Case 7 was 352 pixels x192 pixels (2.200 mm x 1.200 mm) as shown in Figure S5 (Figure 3 in the paper). There is a good virtual slide of this tumor (RS12-7-002.svs), but it does correspond directly to the IR sample. In Figure S5a, the red and green rectangles were for training tumor and nontumor, respectively. The magenta and yellow rectangles were for testing tumor and nontumor, respectively. The orange rectangle is a high lipid region found with the Pearson cross correlation function, and the black rectangle is likely a hole

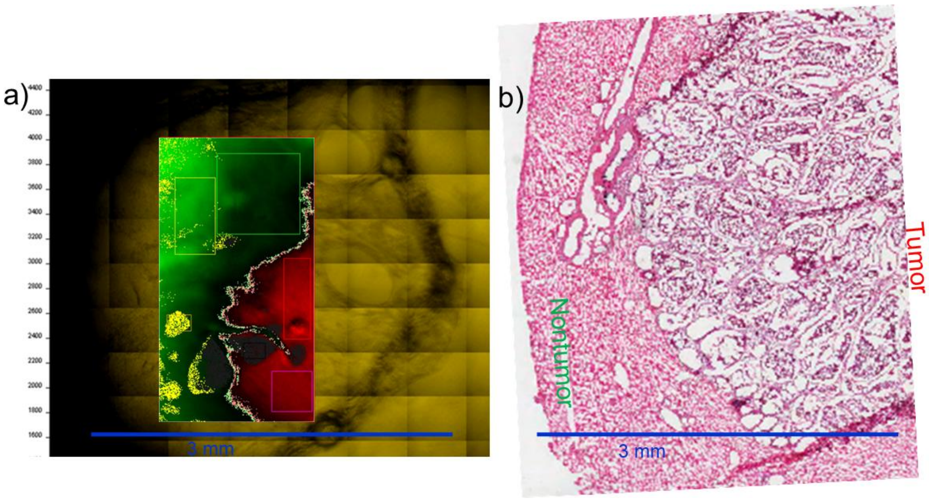

Figure S5. Case 7. a) IR microscope image of the scanning regions with overlay of the decision equation image. The red rectangle is tumor training and the green rectangle is nontumor training, while the yellow rectangle was for nontumor testing and the magenta rectangle for tumor testing. The orange rectangle is a high lipid region, while the black rectangle resembles a hole. b) H&E stain of the same tumor at similar scale, but not the same place in the tissue.

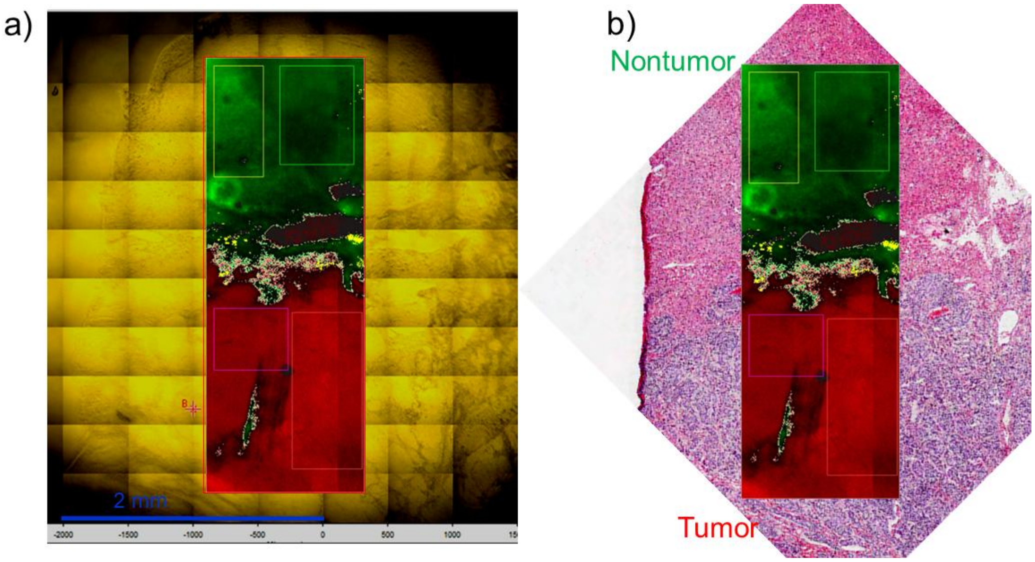

Figure S6. Case 7R has nontumor above and tumor below. a) IR microscope image of the scanning region with overlay of the decision equation image. The red rectangle is tumor training and the green rectangle is nontumor training, while the yellow rectangle was for nontumor testing and the magenta rectangle for tumor testing. b) H&E stain of the adjacent tissue slice at a similar scale and with trackable hole features.

Case 7R was 528 pixels x 192 pixels (3.300 mm x 1.200 mm) and from the same metastatic neuroendocrine carcinoid type tumor. Unlike Case 7, Case 7R had an adjacent H&E virtual slide (RS12-7-005.svs) that followed the hole patterns with fidelity. Figure S6 shows the IR imaging and H&E stain of Case 7R. Again, the red (slightly darker red) and green rectangles

were for training tumor and nontumor, respectively. The magenta and yellow rectangles were for testing tumor and nontumor, respectively. Since Case 7R had such a good virtual slide, Figure S7 was created to show the nontumor and tumor regions at high (40x) magnification which reveals striking differences in cell morphology.

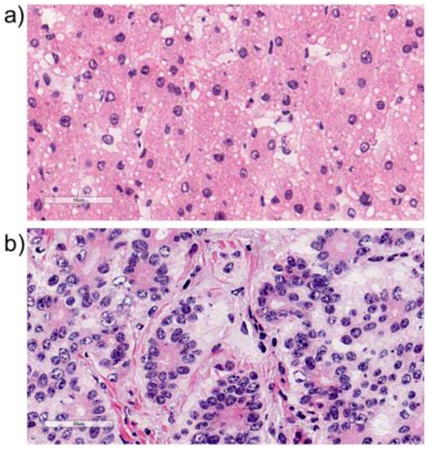

Figure S7. High magnification (40x) snapshots of the a) nontumor and b) tumor regions of Case 7R. The top image is dominated by hepatocyte liver cells and the scale bars are 50 µm.

S1.4 *Case 8.* This case consisted of 4 windows merged into a 352 pixels x 192 pixels (2.200 mm x 1.200) region as shown in Figure S8. The H&E stain was performed on the same piece of tissue underneath the same IR microscope. The H&E stain image used Adobe Photoshop to remove the yellow color cast of the ZnSe optics, to adjust the contrast, and had about four times more spatial resolution than the IR image (although the diffraction limit allows one to do more than ten times better). Note that the IR imaging microscope (Perkin Elmer Spotlight 300) was a Cassegrain system with a pair of hemispherical mirrors allowing a large working region near the sample which is very different than a good optical microscope (such as our Olympus BX40), so spatial resolution was sacrificed in order to get an excellent overlap of the optical and the IR images. The H&E stain can be well overlapped with the IR microscope's image because they both exhibit the circular field of the IR Cassegrain optics. The excellent overlap enabled the merged study of H&E stains and IR imaging as to be discussed later. The H&E stain reveals purple regions which are regions rich in lymphocyte cells

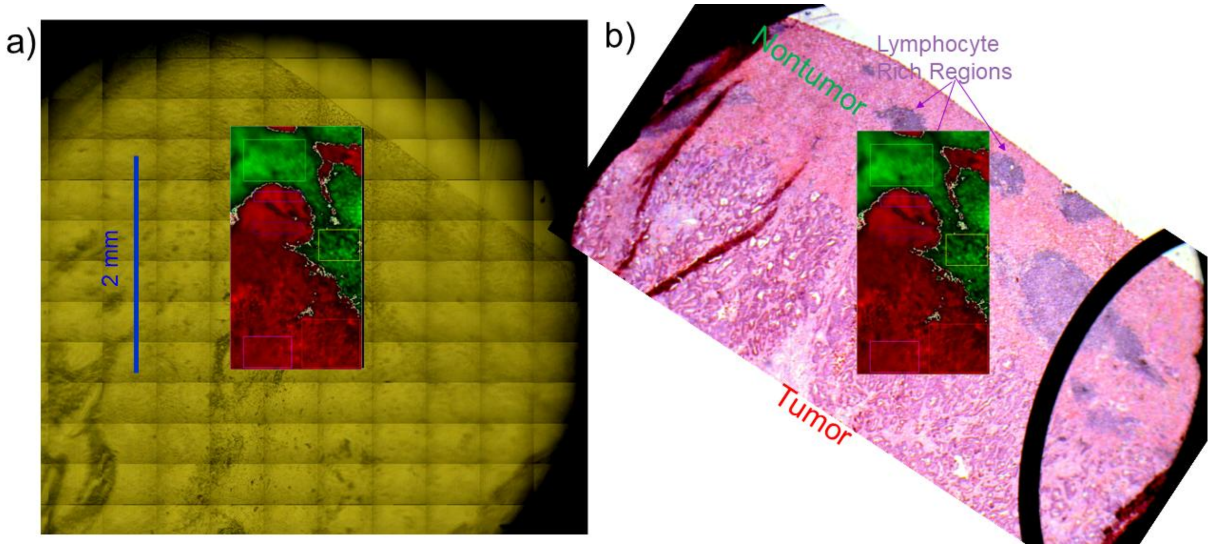

Figure S8. Case 8 has nontumor above and tumor below. a) IR microscope image of the scanning region with overlay of the decision equation image. The red rectangle is tumor training and the green rectangle is nontumor training, while the yellow rectangle was for nontumor testing and the magenta rectangle for tumor testing. b) H&E stain of the exact same slice of tissue. This sample in unique in that there is excellent overlap between the H&E and the IR image allowing the investigation of merging RGB image metrics with IR metrics.

as labeled in Figure S8b revealing a battle ground against cancer. Technically, the lymphocyte regions are not yet tumor and are distinguished as such by the Pathologist. However, they will become tumor if the patient loses this battle, so a surgical oncologist would want to remove these regions if encountered at the surgical margin. It was fastest to scan a region in multiples of 16 pixels to match the detector. As a typical example, the area of 352 pixels x 192 pixels (2.200 mm by 1.200 mm) was scanned in 3 hours and 42 min before the liquid nitrogen cooling ceased. This enables an estimate of the total time required to collect all the data. The training regions for tumor and nontumor are shown with red and green rectangles, respectively, in Figure S8a. The testing regions for tumor and nontumor are shown with magenta and yellow, respectively, in Figure S8a. Rectangles were drawn (purple hued) for testing and training lymphocyte-rich regions as described in the paper. The decision equation employed for the IR scanned region is binary (tumor or nontumor) and did not train with lymphocyte-rich tissues. Interestingly, it shows the lymphocyte-rich regions as tumor, but the user can train the lymphocyte-rich regions as a different group with multiple class approaches.

S1.5 ***Case 9.*** This case was assembled from 5 windows and was merged into a region of 352 pixels x 144s pixels (2.200 mm x 0.900 mm) region as shown in Figure S9. There was a virtual

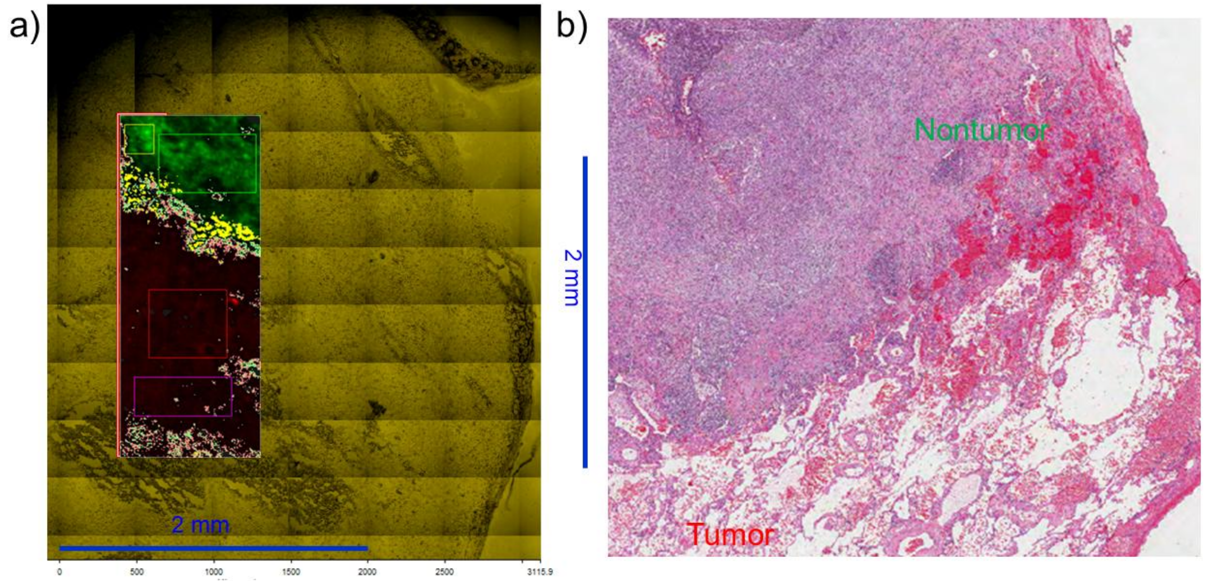

Figure S9. Case 9 has nontumor above and tumor below. a) IR microscope image of the scanning region with overlay of the decision equation image. The red rectangle is for tumor training and the green rectangle is for nontumor training, while the yellow rectangle is for nontumor testing and the magenta rectangle for tumor testing. b) H&E stain of same tumor by a virtual slide (S09-58184-B2.svs), but of a different region.

slide produced from the tumor (S09-58184-B2.svs), but we were not able find adjacent slices or the region analogous to that scanned by the IR imaging. The tumor had many open or white regions (by virtue of the H&E virtual slide , Figure S9b) which looked like dark necrotic regions with open spaces (on the ZnSe window, just at the bottom and below the imaging region in Figure S9a). The experience with Case Samples 1E, 1, 1G, 5, 5A, 5C, 5CH 7, 7R, and 8, gave confidence in the tumor/nontumor decision equations, so training and testing rectangles were

employed. Red and green for training tumor and nontumor, respectively, and magenta and yellow for testing tumor and nontumor, respectively.

S1.6 ***Case 10.*** This case was assembled from 5 windows and was merged into a region of 200 pixels x 240 pixels (1.250 mm x 1.500 mm) as shown in Figure S10. It assays a transition region between tumor and nontumor. The exact IR imaging tissue slice (Figure S10a) was stained with H&E after the imaging (Figure S10b) as with Case 8. This allows very exacting alignment of the

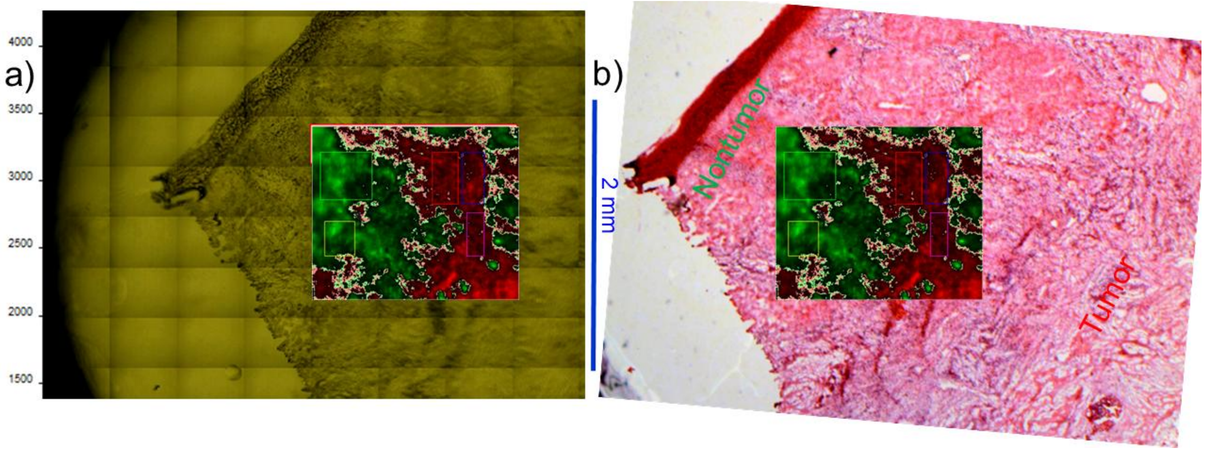

Figure S10. Case 10 has nontumor at left and tumor on the right. a) IR microscope image of the scanning region with overlay of the decision equation image. The red rectangle is for tumor training and the green rectangle is for nontumor training, while the yellow rectangle is for nontumor testing and the magenta rectangle for tumor testing. b) H&E stain of the exact same tissue slice used for IR imaging.

H&E and IR images. In Figure S10a, the red rectangle is for tumor training and the green rectangle is for nontumor training, while the magenta rectangle is for tumor testing and the yellow rectangle is for nontumor testing. The cells in the tumor region adjacent to nontumor, such as in the red rectangle of Figure S10b, look like fibrous stroma cells. These are different than both normal hepatocyte cells and the mature or inner tumor cells. We wonder whether such cells at the boundary of tumor and nontumor transition are diagnostic for a stage of dynamics in the growth of tumor. A training cell was set-up for the fibrous stroma cell region with a blue rectangle in Figure S10a. They may have biological importance like the observation of lymphocyte-rich regions.

S1.7 ***Case 11.*** This case was assembled from 2 windows that were merged into a region of 96 pixels x 368 pixels (0.600 mm x 2.300 mm) as shown in Figure S11. This case turned-out to be all tumor in the IR scan region. We have generally observed that it is easier to get data on the tumor in cancer research than nontumor regions. So, please be advised that a conscious effort is required to get a comparable amount of nontumor data. A red rectangular box for training tumor is rendered in Figure S11 with a magenta rectangle for tumor testing.

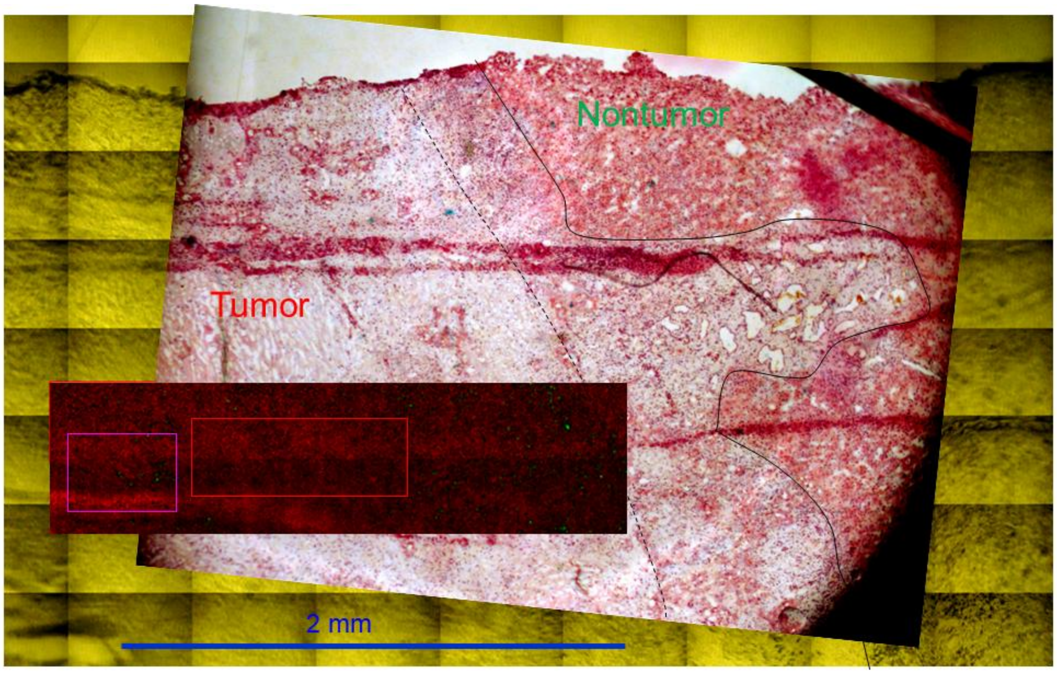

Figure S11. Case 11 is all tumor. The IR microscope image (ZnSe yellow window) of the scanning region with overlay of the H&E stain of the exact same tissue slice used for IR imaging. Then, the decision equation image is overlain. The red rectangle is for tumor training and the magenta rectangle for tumor testing.

S2. Training and Testing Windows. A set of 30 rectangular spectral windows were chosen to be either all tumor or all nontumor as given in Tables S1 and S2. In Figures S1-11, the red and magenta rectangles are tumor windows, while the green and yellow-green rectangles are nontumor windows. Originally, some of the windows were for training and others for testing, but the training schemes changed once case leakage was discovered to be important. These windows were extracted from the full CLM library for the Predictors (spectra at each pixel) and

Table S1. **Training Windows**: Numbers in the name represent the patient, while letters distinguish different samples from the same tumor. In terms of the (i,j) image plane, the upper left corner is (j_{min},i_{min}) and the bottom right is (j_{max},i_{max}).

**Case Sam. Name**	**Tumor Size** (pixels^2)	Tumor X File Training Name	**Tumor Corner Coord.** (j_{min}, i_{min}), (j_{max}, i_{max})	**NT Size** (pixels^2)	**Nontumor X File Training Name**	**Nontumor Corner Coord.** (j_{min}, i_{min}), (j_{max}, i_{max})
1						
5	121x141	Xtrain_T5	(10,50),(150,170)			
7	101x34	Xtrain_T7	(155,150),(188,250)	101x104	Xtrain_NT7	(72,20),(175,120)
7R	191x86	Xtrain_T7R	(105,310),(190,500)	121x91	Xtrain_NT7R	(90,10),(180,130)
8	71x86	Xtrain_T8	(105,280),(190,350)	61x91	Xtrain_NT8	(20,20),(110,80)
9	71x81	Xtrain_T9	(30,180),(110,250)	61x101	Xtrain_NT9	(40,20),(140,80)
10	32x53	Xtrain_T10	(188,169),(240,200)	56x61	Xtrain_NT10	(10,30),(70,85)
11	51x131	Xtrain_T11	(130,20),(260,70)			
1E				96x176	Xtrain_NT1E	(1,1),(176,96)
1G	98x144	Xtrain_T1G	(1,1),(144,98)			
5A				70x40	Xtrain_NT5A	(1,1),(40,70)
5C						
5CH				30x40	Xtrain_NT5C H	(1,1),(40,30)
5D						

Table S2. **Testing Windows:** Numbers in the name represent the patient, while letters distinguish different samples from the same tumor. In terms of the (i,j) image plane, the upper left corner is (j_{min},i_{min}) and the bottom right is (j_{max},i_{max}).

**Case Sam. Name**	**Tumor Size** (pixels^2)	Tumor X File Training Name	**Tumor Corner Coord.** (j_{min}, i_{min}), (j_{max}, i_{max})	**NT Size** (pixels^2)	**Nontumor X File Training Name**	**Nontumor Corner Coord.** (j_{min}, i_{min}), (j_{max}, i_{max})
1						
5	76x91	Xtest_T5	(10,175),(100,250)			
7	51x51	Xtest_T7	(140,290),(190,340)	96x51	Xtest_NT7	(20,50),(70,145)
7R	76x91	Xtest_T7R	(10,305),(100,380)	136x61	Xtest_NT7R	(10,10),(70,145)
8	46x71	Xtest_T8	(20,305),(90,350)	46x61	Xtest_NT8	(130,150),(190,195)
9	41x101	Xtest_T9	(15,270),(115,310)	31x31	Xtest_NT9	(5,10),(35,40)
10	33x22	Xtest_T10	(172,134),(193,166)	41x36	Xtest_NT10	(15,110),(50,150)
11	51x81	Xtest_T11	(40,30),(120,80)			
1E						
1G						
5A				30x40	Xtest_NT5A	(41,1),(80,30)
5C						
5CH				17x38	Xtest_NT5CH	(1,31),(38,47)
5D						

Response (tumor or nontumor index) variables of machine learning routines using the MATLAB programming environment. Training and testing regions were chosen away from tumor-nontumor transitions identified by our Pathologist avoiding the details of where the tumor transition occurs. There were 128,713 spectra identified as training (17.023% of full CLM library) of which 71,174 were tumor and 57,539 were nontumor, while there were 46,151 spectra identified as testing (6.08% of the full CLM library or 36.26% as large as the training set) having 27,272 tumor test spectra and 18,879 nontumor test spectra.

The image plane coordinates in the Tables S1 and S2 can be confusing because they are more related to how we store matrices than how we make two dimensional plots. A brief description is given in Figure S12. The image plane pixels are as denoted by (i, j) on the left-

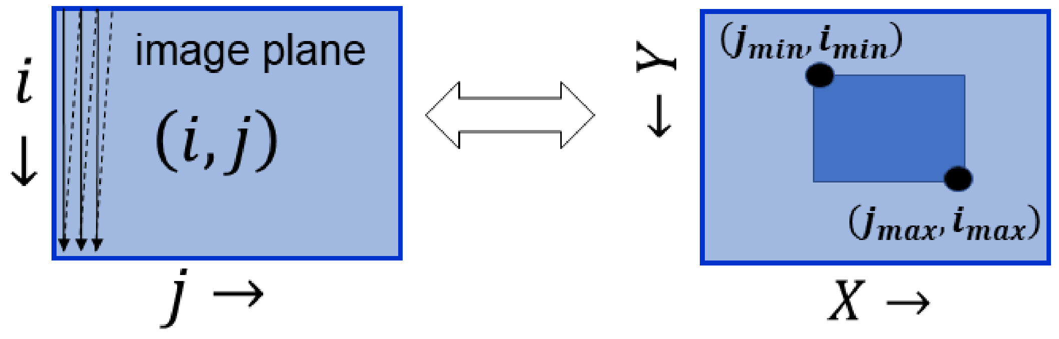

Figure S12. Coordinate systems for drawing training boxes. The (i,j) pixels of an image are reported as (X,Y) coordinates by MATLAB, so it is important to recognize that the pixel "j" value goes with the X coordinate, the "i" pixel value goes with the Y coordinate, but this coordinate gets bigger as it goes lower. The values of j_{min}, i_{min}, j_{max}, and i_{max} go into the MATLAB training program as variables "jmin","imin", "jmax", and "imax".

side of Figure S12 and are rendered as (X, Y) by MATLAB's figure editor on the right-side of Figure S12, so the user needs to know that the "j" column pixel value goes with the X coordinate, the "i" row pixel value goes with the Y coordinate, but this coordinate gets bigger as it goes lower in the image. The values of (j_{min}, i_{min}) and (j_{max}, i_{max}) define a training box with the case tissue sample as shown with black dots on the right side of Figure S12. The values of "j_{min}", "i_{min}", "j_{max}", and "i_{max}" were varied and plotted to verify that the boxes were inside the desired training areas for all sample tissue cases.

The Leave-One-Out (LOO) approach involved a different arrangement than the original windows defined for training or testing. It consists of removing one case from the full dataset, training a decision equation, then testing it on the case that was removed.[5] This gave 7 different trainings avoiding case leakage which could be compared to each other and to any final training with all the data. Take Case 8 as an example. Using the names in Tables S1 and S2, the LOO training for Case 8 involved a catenation of all files except those of Case 8, i.e., the tumor files

Xtrain_T5, Xtrain_T7, Xtrain_T7R, Xtrain_T9, Xtrain_T10,Xtrain_T11,Xtrain_T1G,
Xtest_T5, Xtest_T7, Xtest_T7R, Xtest_T9, Xtest_T10,Xtest_T11

and nontumor files

Xtrain_NT7, Xtrain_NT7R, Xtrain_NT9, Xtrain_NT10, Xtrain_NT5A,Xtrain_NT5CH,
Xtest_NT7, Xtest_NT7R, Xtest_NT9,Xtest_NT10, Xtest_NT5A, Xtest_NT5CH,

comprised the training set (even though many windows were originally intended/named for testing). Then, the testing was done with all of the Case 8 files, i.e., the tumor files

Xtrain_T8, Xtest_T8

and the nontumor files

Xtrain_NT8, Xtest_NT8,

so that testing also included windows originally intended/named for training.

S3 Full Library Loading. The names, sizes, stains, and Perkin Elmer datablock files of the tissue sample cases of the CLM library are given in Table S3. The samples are first labeled by the Case # indicating a different patient. Then, many cases had multiple tissue sample regions which were distinguished by letters as shown in the 1st column of Table S3. Tissue sections

Table S3. Names, Sizes, Stains, and Datablock files of Tissue Sample Cases. Numbers in the name represent the patient, while letters distinguish different samples from the same tumor.

**Name**	**Size** (pixels^2)	**Size** (mm^2)	Tumor or Nontumor	**H&E Stain**	**MATLAB Input File Names (*.fsm files)**
1	96x176	0.600x1.094	both	RS12-001-2.svs	e#ac, #=1-66
1E	96x176	0.600x1.094	nontumor	RS12-001-2.svs	o#ac, #=1-66
1G	96x144	0.600x0.900	tumor	RS12-001-2.svs	r#ac, #=1-54
5	352x240	2.200x1.500	tumor	our microscope	ac_case5_#, #=1-5
5A	528x160	3.305x1.000	both	our microscope	Case5A_#, #=1-5
5C	352x240	2.200x1.500	both	our microscope	Case5C_#, #=1-5
5CH	352x48	2.200x0.300	both	our microscope	Case5CH
5D	192x352	1.200x2.200	tumor	our microscope	Case_5D_#,#=1-4
7	352x192	2.200x1.200	both	RS12-7-01.svs*	ac_case7_#, #=1-4
7R	528x192	3.300x1.200	both	RS12-7-01.svs*	ac_case7R_#, #=1-6
8	352x192	2.200x1.200	both	our microscope	ac_case8_#, #=1-4
9	352x144	2.200x0.900	both	S09-58184-B2.svs	ac_case9_#, #=1-5
10	200x240	1.250x1.500	both	our microscope	ac_case10_#, #=1-5
11	96x368	0.600x2.300	tumor	our microscope	ac_case11_#, #=3,4

*There are many other virtual slides for this case including RS12-7-02.svs, RS12-7-03.svs, RS12-7-06-HE.svs, RS12-7-19-HE.svs, as well as trichrome stains RS12-7-03-TRICHROME.svs, RS12-7-07-TRICHROME.svs, and RS12-7-11-TRICHROME.svs.

adjacent to the one used for IR imaging were sometimes sent out to a virtual slide facility [OSU Wexner Medical Center JML Molecular Laboratory @ Polaris, now moved onto OSU campus] and we have virtual slides for Cases 1, 7, and 9. Virtual slides are made by automated scanning and stitching of high resolution rectangular regions producing an imaging data file allowing a user to zoom in or out on a computer screen, annotating or collecting snapshots, as one would on a microscope – but more rapidly and conveniently. Other sections were stained by us on the same sample after IR scanning on our own optical microscopes, including Cases 5, 8, 10 and 11. This allows the possibility of excellent overlapping of the IR image and H&E stain, if the H&E staining process did not move the frozen section too much. While the in-house stains are generally of lower spatial resolution than a virtual slide, they give rise to the possibility of a

combination or merger of IR imaging and H&E staining, *i.e.,* the coloring of an optical microscope image by means of the IR biomolecular signatures.

MATLAB programs were written to take the data formats of the instrument - however they were measured and put them in a form for machine learning. Datablock format is shown in Figure 1c in the paper. Note the arrows within the blue image plane of Figure 1c that indicate how they are placed into machine learning format (Figure S13a) which also allows stacking into merged files (Figure S13b) for combined use with machine learning programs. In the input

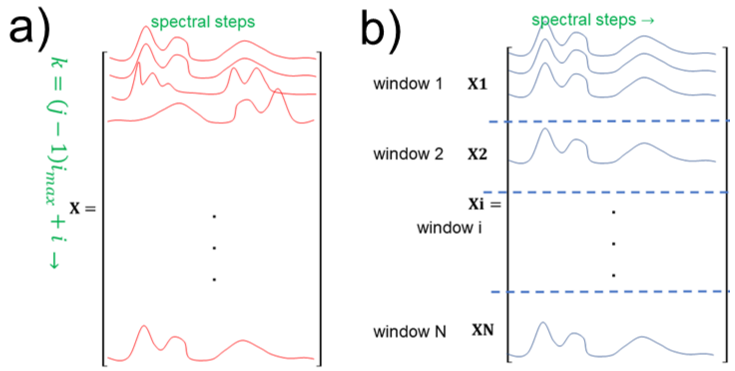

Figure S13. a) The three-dimensional format of Perkin Elmer IR imaging windows (Figure 2c) must be converted to Predictor file format with one spectrum per row while keeping knowledge of the corresponding image pixels. Different subsets from different case tissue samples can be catenated to make a bigger Predictor file where the user must maintain knowledge of the corresponding image and its' pixel.

Predictor files ("X" file in Figure S13a), there is one spectrum per row as associated with a particular image pixel in a particular case tissue sample. If we take pixel coordinates, (i, j), in image space (see Figure 2c), then the row, k, in that Predictor file is

$$k = (j-1)i_{max} + i \quad , \qquad \text{(s1)}$$

such that every pixel in the image file is correlated with a row in a Predictor file. Each case tissue sample Predictor file of Table S3 is stacked in the manner illustrated in Figure S13b. Multiple windows (of different case tissue samples, X1, X2, …XN in Figure 13b) are catenated, i.e., added as matrices to the bottom of each successive window. The user maintains knowledge of the corresponding tissue sample and case and image pixel coordinates.

The MATLAB program in the box below makes a raw (no preconditioning) Predictor file (X file) of everything in the CLM library. One ends up with a Predictor matrix (X file) called "X" which is 756,096 x 1626 in which each row is a spectrum and there are 1626 steps in each IR spectrum. Also included are the pixel numbers (sizes) of each case sample with MATLAB names "nx#" and "ny#" where # is the case and sample number. Finally, the wavenumbers of the spectral steps are stored in the MATLAB variable "nu". Loading the whole raw CLM library requires ~2.3 min to load with a PC (Dell Precision 3630 PC with 128 Gb RAM). After running

the training and testing sets, the results ended using 31 Gb (out of 128 Gb) of RAM where 17 Gb are due to the operating system and MATLAB.

```
% ***** Make a Predictor X file of the Whole CLM Library ***************
tic % time the process
load xfilecase1.mat; disp('X1 loaded'); % case 1
load xfilecase5.mat; disp('X5 loaded'); % case 5
load xfilecase7.mat; disp('X7 loaded'); % case 7
load xfilecase7R.mat; disp('X7R loaded'); % case 7R
load xfilecase8.mat; disp('X8 loaded'); % case 8
load xfilecase9.mat; disp('X9 loaded'); % case 9
load xfilecase10b.mat; disp('X10 loaded'); % case 10
load xfilecase11.mat; disp('X11 loaded'); % case 11
load xfilecase1E.mat; disp('X1E loaded'); % case 1E
load xfilecase1Gb.mat; disp('X1G loaded'); % case 1G
load xfilecase5A.mat; disp('X5A loaded'); % case 5A
load xfilecase5C.mat; disp('X5C loaded'); % case 5C
load xfilecase5CH.mat; disp('X5CH loaded'); % case 5C
load xfilecase5D.mat; disp('X5D loaded'); % case 5D
clearvars -except nu X1 nx1 ny1 ...
                   X5 nx5 ny5  ...
                   X7 nx7 ny7  ...
                   X7R nx7R ny7R  ...
                   X8 nx8 ny8 ...
                   X9 nx9 ny9 ...
                   X10 nx10 ny10  ...
                   X11 nx11 ny11  ...
                   X1E nx1E ny1E  ...
                   X1G nx1G ny1G  ...
                   X5A nx5A ny5A  ...
                   X5C nx5C ny5C  ...
                   X5CH nx5CH ny5CH ...
                   X5D nx5D ny5D;
X=cat(1,X1,X5,X7,X7R,X8,X9,X10,X11,X1E,X1G,X5A,X5C,X5CH,X5D);
toc
```

S4 Spectral Preconditioning, Holes, and Lipids. Four preconditioning options for the CLM library were explored: i) raw (no preconditioning), ii) baseline-corrected, iii) baseline-corrected and ratioed, and iv) baseline-corrected and normalized. Noticing that the baseline of the average CLM spectrum is not zero (see Figure 1d in the paper), it is clear that scattering effects are important in spectral tissue imaging work. Baseline correction was needed as a starting strategy for accentuating absorption and diminishing scattering. Furthermore, many individual spectra in the CLM library may not look like the average protein dominated spectra. Sometimes tissue sections have holes due to preparation trouble or natural structures like venules, arterioles, or bile ducts. Likewise, many applications will not measure an absolute spectrum (as with a 3 μm thick slab of tissue). So, this section develops strategies for more broad applicability. One does better in distinguishing cancer by not correcting for baseline, so take this processing as an effort for more broadly applicable results. Also, the search for other biomolecules is facilitated by baseline

corrected spectra, so baseline correction is needed and is essential to make the ratioed or normalized options work.

Baseline-Correction. Non-zero baselines are found throughout the CLM library due to scattering effects. Our signal is technically extinction (absorption and scattering), but the absorption components are most apparent. Since the many cells in tissue sections are being observed at a slightly subcellular resolution, it is reasonable that the changes in index of refraction that allow cell structures to be observed under the microscope (membrane outlines for cell size, nuclei, and vacuoles) will also be different in tumor versus nontumor tissue. Consequently, we separate-out some scattering effects by virtue of baseline correction using eight points that are in regions devoid of absorption signal and that span the spectral range: 3956, 3726, 2650, 2450, 2110, 1780, 890, and 800 cm^{-1}. A spline fit is accomplished with the moving average default method as shown in Figure S14. The effectiveness of the "moving average, eight-point smooth method" was demonstrated by observation of a flat baseline at zero for the average spectrum of the 14 case tissue samples. Please note that any points chosen for the spline fit become zero and do not contribute to the decision equation value. It is worth considering if the scattering information should be discarded because it contains interesting information regarding tumors. Three metrics were extracted after baseline correction:

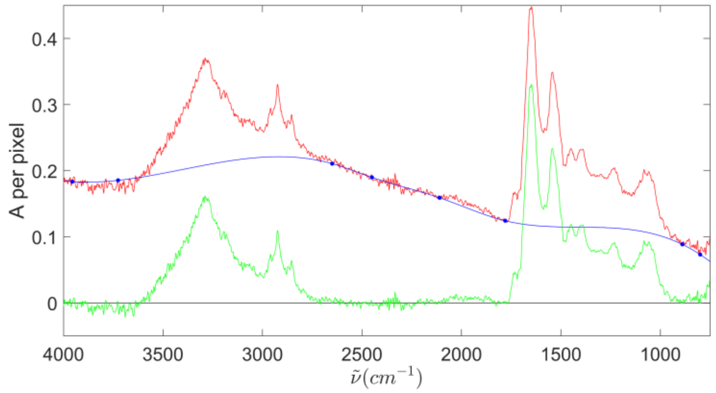

Figure S14. A single pixel IR raw spectrum (red) shows the eight wavenumbers chosen (blue dots) for a spine fit (blue trace). The bottom shows the result of subtracting the spline fit where the baseline corrected curve is shown as green.

- sum of absorbances after baseline correction
- norm of the baseline corrected spectrum
- Pearson cross correlation with chosen target (lipid).

Histograms of the three baseline-correction parameters are plotted in Figure S15a for one Case 7 tissue sample. This case tissue sample was particularly interesting because there are multiple distributions in each of these baseline-correction metrics. The top histogram (digital sum for Integrated Absorption) shows at least 4 distributions revealing different densities of tissue in different regions. The middle histogram labelled "Norm" shows even more detail (five distributions) and is useful for comparing to IR measurements on samples other than 3 μm tissue sections. The "sum" and "norm" metrics both show very low value distributions that are correlated with holes. The bottom histogram shows that there are two distributions of lipid. Generally, in this liver tissue, the higher distribution is for nontumor, and the lower one is for tumor. Clearly, searches for cross correlation with chosen calibrant molecules can facilitate understanding the biochemistry of these tissues. It was surprising to us that baseline metrics

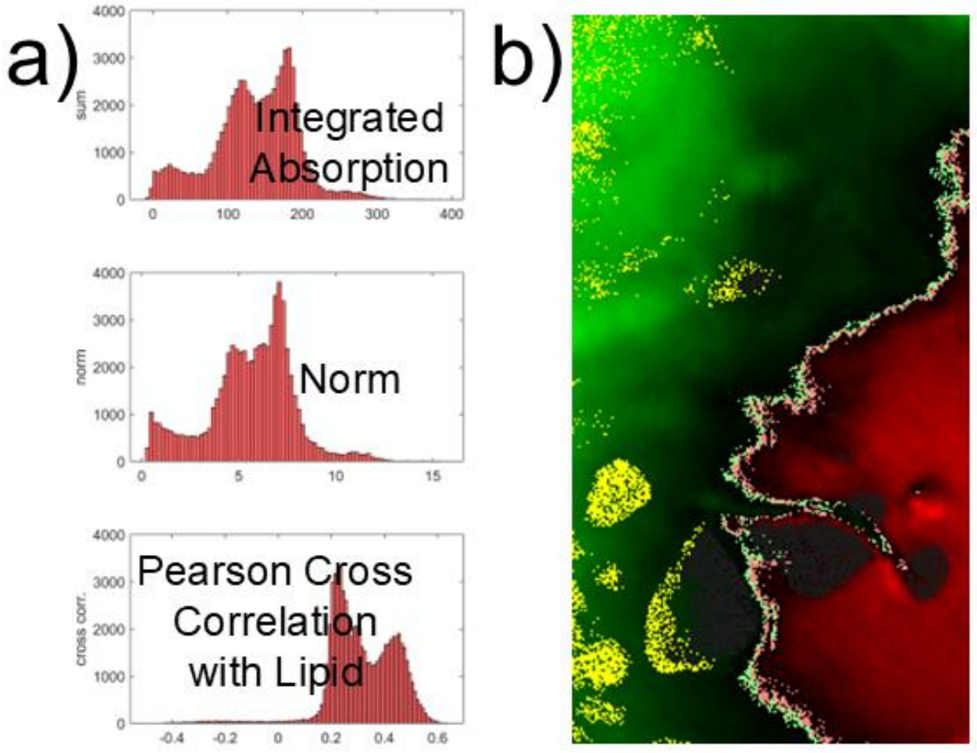

Figure S15. a) Histograms of the three baseline-correction metrics for Case 7: sum of absorbance after baseline-correction, the norm after baseline-correction, and the cross-correlation with lipid. Note the these are not simple distributions. b) Decision equation imaging of Case 7 (red for tumor and green for nontumor). The transition is highlighted with light green on the nontumor side ($-0.5 < d_k < 0$) and light red on the tumor side ($0 \le d_k < +0.5$). Holes are plotted with dark gray and high lipid correlation is plotted with yellow.

themselves are useful for discriminating against tumors, although not anywhere near as good as the IR absorption of vibrational features.

Normalization After Baseline-Correction. The raw and baseline-correction options are not of wide and general use because applications are unlikely to measure a signal directly comparable to a 3 μm thick slab of tissue. Consider the design of an IR absorption probe: different operators will push the sensors into the tissue in different amounts making different intensities of absorption. Only relative measurements are important for such probes. Euclidean normalizations were performed and also stored if needed as metrics. If S_j is the baseline-corrected spectrum (green trace of Figure S14), then the Euclidean norm is

$$\sqrt{\sum_j^{ns} S_j^2}, \qquad \text{(s2)}$$

where ns is the number of steps in the spectrum. Next, methods are discussed to search the CLM library for holes and target biomolecules.

Holes. Holes may occur in these tissue slices due to tearing in preparation or as arterioles, venules, bile ducts, or capillaries. Given baseline correction, then the holes have less tissue than normal, so low values of the integrated area of the spectrum after baseline correction can indicate holes. Noting that tissue fluids can leak into these regions, one might also look for a spectrum not dominated by protein. If there is a low value tail in the histogram of the integrated area, then there are likely holes to worry about in the data. Thresholds are set empirically by trial-and-error and holes are plotted with black or a dark grey color.

Biomolecules. While the average tissue spectrum is dominated by protein, there are bound to be places where other biomolecules are evident. Unfortunately, IR searches on commercial spectrometers for biomolecules are generally good, but proprietary. We can get a graphic, but not a digital file of the matches. Consequently, we developed our own programs for identifying holes and similarity to known biomolecules. The yellow in Figure 2a in the paper is due to lipid with the following strategy. We are curious about other lipids in the CLM library because steatosis[6-8], or fatty liver disease, is an important condition unto itself and an important factor to consider in judging the risks of performing liver tumor resection. Noting that the Pearson cross correlation is defined as the linear correlation between two spectra as normalized by the standard deviations of those spectra, code was written to search for lipids. A reference lipid spectrum is chosen (usually by scaled subtraction of protein in different tissue spectra) and a similarity (scaled like a probability) is calculated of each measured CLM spectrum to that of the chosen molecule. Taking G_j as the target molecule spectrum and S_j as the measured and baseline-corrected CLM spectrum, the Pearson cross correlation [https://en.wikipedia.org/wiki/Pearson_correlation_coefficient] is defined as

$$r_{S,G} = \frac{\sum_{j=1}^{ns}(S_j G_j - ns\bar{S}\bar{G})}{(ns-1)\sigma_S \sigma_G}, \qquad \text{(s3)}$$

where $\bar{S}$, $\bar{G}$, σ_S, σ_G are means and standard deviations obtained with MATLAB functions (“mean” and “std”). The cross correlation, $r_{S,G}$, ranges from -1 to +1 where a high value indicates similarity to the target molecule spectrum. The user sets a threshold for finding the molecule (lipid in this work). For example, cross correlation greater than 0.5 might be plotted in yellow.

S5 Extra Results

S5.1 Decision Equation Output. The decision equations data [three spectra and a constant, i.e., $\overline{Train}_j$, $\sigma_{Train\,j}$, β_j, and b of Equation (2) in the paper] are offered on the left side of an Excel file called “LOO_decision_equations_CML_check.xlsx” for each of the Leave-One-Out Decision Equation Trainings. Also, the average tumor and nontumor spectra are given on the right side so Decision Contribution Spectra can be computed. Note that the eight points of the spline fit baseline correction get zeroed and values at these points are not given in the Excel file. These results can be used to test spectra that have the same spline fit baseline-corrected with normalization. They must also have the same spectral format as this work: spectral resolution of 4 cm^{-1}, in a spectral range 4000-750 cm^{-1}, with 1626 steps of 2 cm^{-1}.

S5.2 ***Peak Ratio Metrics of Previous Work*** . Our early efforts on metastatic liver cancer frozen section spectra used peak intensity ratio metrics as learned from Bhargava and coworkers[9] on a different cancer type. Noting that their work also had some summed metrics, we extracted 36 peak ratios shown in Table 2. Peak ratios are useful because they are independent of tissue slice thickness. We call this set the "Bhargava36" and note that 27 of these metrics are ratioed to the amide II band near 1544 cm^{-1}. The amide I band can saturate if the frozen sections are 5 µm thick or more, hence the use of 3 µm sections in this work. Ratio to the amide II band likely conveys more stability to the results by avoiding or reducing amide I saturation effects. A predictor file was created with 36 columns of absorbance ratios from Table S4. Upon SVM

Table S4. Thirty-Six Peak Ratio Metrics, i.e. the "Bhargava36".

metric name	Ratio	metric name	Ratio	metric name	Ratio	metric name	Ratio
b_1	I_{966}/I_{1544}	b_{10}	I_{1236}/I_{1544}	b_{19}	I_{1016}/I_{1080}	b_{28}	I_{1516}/I_{1236}
b_2	I_{1012}/I_{1256}	b_{11}	I_{1278}/I_{1544}	b_{20}	I_{1032}/I_{1080}	b_{29}	I_{1064}/I_{1544}
b_3	I_{1034}/I_{1544}	b_{12}	I_{1502}/I_{1544}	b_{21}	I_{1020}/I_{1034}	b_{30}	I_{1162}/I_{1544}
b_4	I_{1062}/I_{1544}	b_{13}	I_{1516}/I_{1544}	b_{22}	I_{1050}/I_{1544}	b_{31}	I_{1240}/I_{1544}
b_5	I_{1080}/I_{1544}	b_{14}	I_{1536}/I_{1544}	b_{23}	I_{1080}/I_{3290}	b_{32}	I_{1396}/I_{1544}
b_6	I_{1114}/I_{1544}	b_{15}	I_{1588}/I_{1544}	b_{24}	I_{1164}/I_{1080}	b_{33}	I_{1520}/I_{1544}
b_7	I_{1158}/I_{1544}	b_{16}	I_{1654}/I_{1544}	b_{25}	I_{1400}/I_{1390}	b_{34}	I_{1528}/I_{1544}
b_8	I_{1170}/I_{1544}	b_{17}	I_{3290}/I_{1544}	b_{26}	I_{1426}/I_{1450}	b_{35}	I_{1668}/I_{1544}
b_9	I_{1206}/I_{1544}	b_{18}	I_{3292}/I_{1544}	b_{27}	I_{1450}/I_{1544}	b_{36}	I_{3342}/I_{1544}

training and testing using the baseline-corrected option, a training error of 1.751% and a testing error of 1.770% were obtained (case leakage model). Now this is not as good as the full spectra results from the four options, but it is exceptionally good. It demonstrates that a reduced set of wavelengths can be used.

Twenty-eight peak ratios were added to the "Bhargava36" as shown in Table S5. The

Table S5. The "Coe-Bhargava64" set added 28 more peak ratios to the "Bhargava36" of Table 2.

metric name	Ratio	metric name	Ratio	metric name	Ratio	metric name	Ratio
b_{37}	I_{1744}/I_{1244}	b_{44}	I_{2874}/I_{2854}	b_{51}	I_{1588}/I_{1548}	b_{58}	I_{1650}/I_{1548}
b_{38}	I_{1744}/I_{1162}	b_{45}	I_{1120}/I_{1020}	b_{52}	I_{1520}/I_{1548}	b_{59}	I_{1656}/I_{1548}
b_{39}	I_{1024}/I_{1080}	b_{46}	I_{2924}/I_{1544}	b_{53}	I_{1160}/I_{1548}	b_{60}	I_{1662}/I_{1548}
b_{40}	I_{1172}/I_{1154}	b_{47}	I_{1516}/I_{1582}	b_{54}	I_{2916}/I_{1548}	b_{61}	I_{1670}/I_{1548}
b_{41}	I_{2854}/I_{2962}	b_{48}	I_{1536}/I_{1544}	b_{55}	I_{1600}/I_{1548}	b_{62}	I_{1676}/I_{1548}
b_{42}	I_{1080}/I_{1244}	b_{49}	I_{1080}/I_{1548}	b_{56}	I_{1620}/I_{1548}	b_{63}	I_{1686}/I_{1548}
b_{43}	I_{1744}/I_{1548}	b_{50}	I_{1030}/I_{1080}	b_{57}	I_{1632}/I_{1548}	b_{64}	I_{1696}/I_{1548}

combined set of Tables S4 and S5 is herein called the "Coe-Bhargava64" and the SVM training and testing errors were 1.518% and 1.425%, respectively. So, this was better than the "Bhargava36", and not as good as the full ratioed option.

An attempt was made to reduce the number of peak ratios that were needed. A selection of 20 peak ratio metrics was made from the "Coe-Bhargava64" set and applied to breast cancer metastatic to the liver.[10] The 20 metrics are given in Table S6. The SVM training and testing were performed on the baseline-corrected CLM library giving errors of 7.181% and 6.054% for training and testing, respectively. While this effort is good (94% correct in testing) and may have worked well for breast cancer metastatic to the liver, it is considerably worse than the "Bhargava36" and "Coe-Bhargava64" peak ratio sets on the CLM data set.

Table S6. The "Coe_L20" were a subset selected from the "Coe64". Both the names from the paper and Tables 8 and 9 are given.

metric name	Ratio	metric name	Ratio	metric name	Ratio	metric name	Ratio
L_1, b_{43}	I_{1744}/I_{1548}	L_6, b_{54}	I_{2916}/I_{1548}	L_{11}, b_{19}	I_{1016}/I_{1080}	L_{16}, b_7	I_{1744}/I_{1162}
L_2, b_{37}	I_{1744}/I_{1244}	L_7, b_{45}	I_{1120}/I_{1020}	L_{12}, b_{59}	I_{1252}/I_{1544}	L_{17}, b_{23}	I_{1080}/I_{3290}
L_3, b_{64}	I_{1742}/I_{1256}	L_8, b_{46}	I_{2924}/I_{1544}	L_{13}, b_{39}	I_{1024}/I_{1080}	L_{18}, b_{58}	I_{1662}/I_{1548}
L_4, b_{53}	I_{1160}/I_{1548}	L_9, b_{48}	I_{1080}/I_{1548}	L_{14}, b_{42}	I_{1080}/I_{1244}	L_{19}, b_2	I_{1012}/I_{1256}
L_5, b_{28}	I_{1516}/I_{1236}	L_{10}, b_{38}	I_{1744}/I_{1162}	L_{15}, b_{47}	I_{1516}/I_{1582}	L_{20}, b_{30}	I_{1162}/I_{1544}

A summary of the SVM training and testing results with peak ratios is given in Table S7. Clearly, there is great potential for reducing the number of wavelengths and linear SVM represents a good way to ascertain the quality of methods.

Table S7. Summary of training and testing errors for different peak ratio methods using the baseline-corrected option of the CML library. For training, there were 124,199 total spectra of which 68,688 are tumor and 55,511. For testing, there were 46,151 total of which 27,272 are tumor and 18,879 are nontumor.

**Method**	**Training Error (%)**	**Testing Error (%)**
full spectrum ratioed	0.319	0.472
Coe-Bhargava64	1.518	1.426
Bhargava36	1.751	1.770
Coe_L20	7.181	6.054

S5.3 ***Tumor/Nontumor Transitions.*** Linear scaled (standardized) SVM Decision equation values from -0.5 to 0 defined the nontumor side (using light green) and from 0 to +0.5 defined the tumor side (using light red) as shown in Figure S16 which is a zoomed region of Case 10. When using scaled SVM, the region of decision equation values from -1 to +1 represents a width of two standard deviations of the scaled training set, i.e., ± one standard deviation about the decision boundary in the scaled SVM space. Each pixel is 6.25 μm x 6.25 μm, so a boundary region that is 4 pixels wide is about 25 μm which is a subcellular dimension for liver hepatocyte cells. While the thickness varies from case to case, it looks like it is often defined by our criteria on a single cell scale. There is an average tendency for light red to be towards inner tumor and light green to be towards inner nontumor, but there is also significant mixing. If one were interested in tracking the biomolecules at the transition, the IR spectra could be computed for decision equation values from -0.5 to +0.5 and then compared to the nontumor and tumor average spectra.

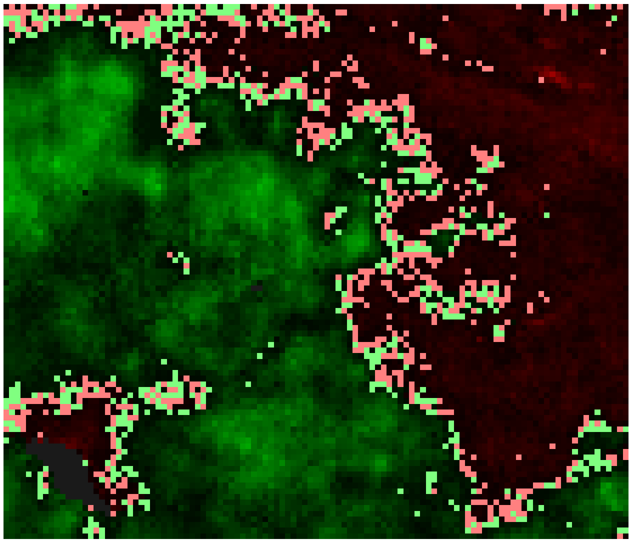

Figure S16. Zoom into the transition region of Case 10 where green is nontumor and red is tumor. For the tumor/nontumor transition, light green indicates decision equation values from -0.5 to 0, while light red indicates values from 0 to +0.5.

S5.4 ***K-Means Clustering with Decision Equation Values***. After having obtained a training that tests well, one can obtain a decision equation value and a classification at every pixel in the full CLM library (756,096) as compared to the 128,713 for window training, i.e., a 5.9-fold increase in the database. Unsupervised studies [requiring only the Predictor file (X file)] are usually performed before supervised techniques [requiring both Predictor (X file) and Response files]. However, it is interesting to go back to the common unsupervised methods of K-Means Clustering (k-means) and principal component analysis (PCA) with the perspective that these can be evaluated in terms of SVM decision equation values. K-means is useful to us as a way of checking the details of our algorithms. It might also be used with our SVM decision equation results for more incisive analysis, as we have found with PCA. First, as previously described, the decision equations values were used to image the IR windows of this work, but they can also be used to identify the transition between tumor and nontumor.

A k-means clustering analysis was done on the full CLM library with k (the number of clusters) set at 25. The method takes the Predictors and partitions them into k clusters in which each pixel goes to the cluster with the nearest mean (cluster center or centroid is close to the average spectrum of the cluster). K-means is not the most rigorous of such methods, but it is simple and useful with large data sets such as the CLM. We found it very useful for identifying clusters that were inappropriately prepared, for example during baseline correction. This guided the change from a parabolic baseline-correction to an eight-point moving average approach. More importantly, it can be used to isolate molecules of interest. A 25 cluster, k-means analysis was performed on the full, baseline-corrected CLM library and the centroid spectrum of each cluster is shown in Figure S17a with color coding for the clusters. Recalling that centroids are very close to the average spectrum of all members of the cluster, note that each of the 25 clusters has a unique and different IR spectrum and that there are biochemical molecular differences in

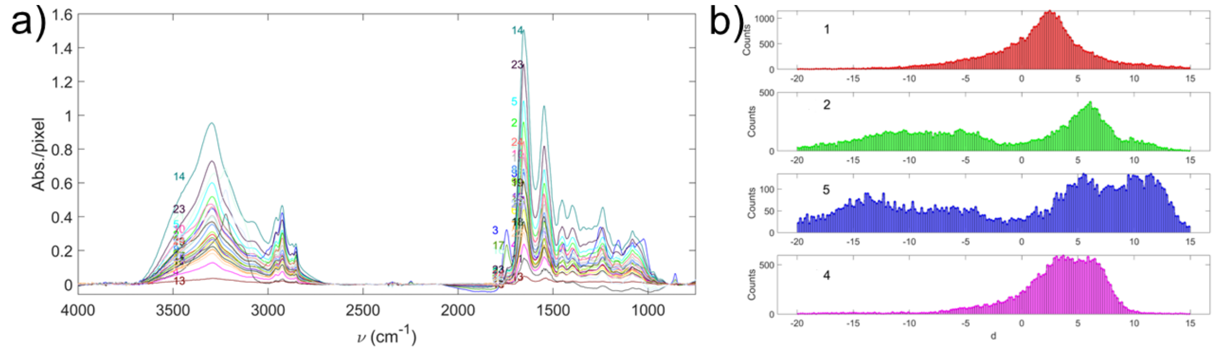

Figure S17. a) 25 centroids of k-means clustering for the full CLM library. b) Decision equation histograms of the #1, #2, #5, and #4 clusters where tumor has positive decision equation values and nontumor is negative..

all the spectra. Clusters #3, #16, and #17 have high amounts of ester-linked lipid (vibration at 1744 cm^{-1}) so these could be used to isolate high lipid spectra. It would be useful and convenient if many of the clusters could be classified as pure tumor or pure nontumor. For instance, one can calculate the average and standard deviation of the decision equation value for each cluster (see Table S8). Only a few of the clusters (nontumor #3, #16, #17 and tumor #10, #11, #21, #25) are mostly one or the other as judged by the average decision eq. magnitude being considerably bigger than the standard deviation. Most of the clusters have evidence of both tumor and

nontumor as shown with the histograms of clusters #1, #2, #5, and #4 in Figure 12b. There may be some uses of clusters that are all tumor (or all nontumor), but 18 out of 25 clusters show both tumor and nontumor components and are not useful for the tumor-nontumor distinction. Table S8 gives the number of pixels (spectra), the average decision equation value, and the standard deviation of decision equation values for each cluster. Notice how only a few clusters are predominantly tumor or nontumor and note that many have standard deviations bigger than the mean.

Like our previous work on breast cancer,[10] the high lipid spectra of clusters # 3, #16, and #17 have a CN triple bond stretch peak at 2247.6 cm^{-1} which is unexpected naturally. Taking a scaled difference between cluster # 3 and the more typical cluster #1, a spectrum is isolated in Figure S18 (blue trace) with a small and narrow band at 2247.1 cm^{-1}. A search with Perkin Elmer Spectrum software and the FDM (Fiveash Data Management) library found a match with Loctite Duro superglue (red trace in Figure 13). Superglue has the polymer

Table S8. K-means clustering on full CLM library

Cluster	#	Avg. D	Std D
1	40525	1.56	5.07
2	23039	-3.39	10.31
3	10595	-59.46	12.99
4	24194	2.60	5.92
5	12089	-0.58	10.84
6	97918	1.57	3.62
7	46232	-2.58	5.95
8	53229	-0.26	7.06
9	59384	1.83	5.22
10	7619	8.66	5.03
11	2181	6.38	2.12
12	50334	-1.27	8.24
13	18201	-0.08	7.06
14	692	6.49	17.94
15	59266	1.57	4.52
16	8361	-29.88	13.70
17	6301	-24.17	13.30
18	88944	2.23	3.36
19	15125	5.92	6.72
20	14774	-1.45	8.85
21	2296	12.94	2.71
22	70705	3.43	4.04
23	5460	-1.27	13.57
24	36181	-2.14	8.76
25	2451	9.45	2.22

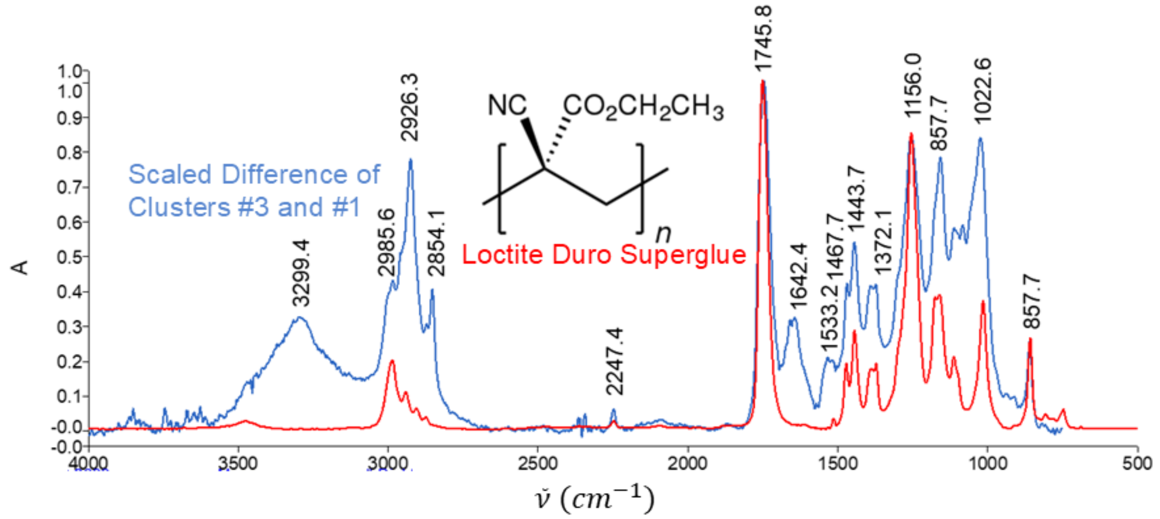

Figure S18. Best match from the FDM library with the difference of high lipid cluster and a typical cluster (blue) as determined with the Perkin Elmer software giving a 0.846 matching search score with Loctite Duro Superglue which has a CN triple bond.

formula of ethyl 2-cyanoacrylate (see inset of Figure S18). We are not aware of this polymer occurring naturally in liver tissue, but similar polymers (cyanoacrylates) are used by surgeons as tissue glue[11, 12]. Searching routines of the CLM case tissue images found that the CN triple bond stretch peak (cyanoacrylate spectrum) occurs in 25,258 spectra of the 756,096 CLM library which is 3.34%. The cyanoacrylate signatures are seen in 6 out of 7 cases and distributed in a biologically meaningful way. They are not due to use of cyanoacrylate glue to secure the tissue section samples with a thin uniform coating. Is cyanoacrylate a natural minority lipid? Might chemotherapy enable such triple bonds in lipids? Is there some subtle aspect of sample preparation that adds cyanoacrylate? More work is needed.

S5.5 *Principal Components with Decision Equation Values.* Principal component analysis (PCA) can reduce the dimensionality of a large data set while maintaining distinctions of interest. It determines a linear and orthogonal set of spectral-like vectors called the principal components (PCs) by means of single-value decomposition in the most rigorous implementation. Ultimately, each datapoint (pixel or row in the X file) can be assigned a score for each principal component. The principal components (PCs) are generally sorted from largest to smallest contributions and users often select a small subset of PCs making the largest contributions to represent the data (reducing dimensionality). The top 11 principal components (PCs) on the full CLM library (706,596 spectra) are shown in Figure S19 as accomplished without mean centering such that the first PC (PC1) is very close to the average spectrum. PCA is very much an analysis of changes from average, so in this analysis one looks to PC2-PC11 for chemical specificity. For example, PC2 has ester-linked lipid characteristics, PC3 and PC4 have protein changes in the amide I and II band regions, PC7 and PC10 have lipid related signatures, PC8 and PC9 are dominated by background CO_2 gas, and PC11 is dominated by glycogen which is a cellular energy source. Clearly PC scores can indicate biochemical changes, however PCs also indicate changes in the amounts of particular biomolecules, so the numbering of

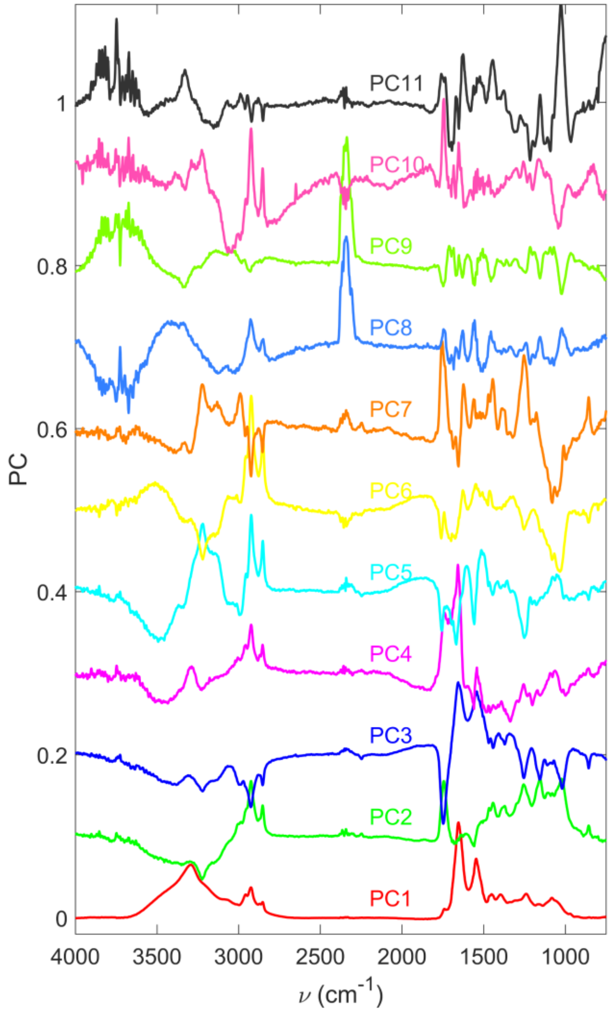

Figure S19. The 11 most important PCs of the full CLM library from PC1 at the bottom (red) to PC11 at the top (black) with an offset for better viewing.

the PCs is not biochemically dominated, and the user might have to search to higher numbers of PC for desired chemical changes. Also, at some point, the eigenvectors become dominated by noise and are not useful for representing the signal, but this point is well beyond PC11 as shown in Figure S19.

The training decision equation for the baseline-corrected option (based on 128,713 spectra) with 99.7% testing accuracy was used to classify all points in the full CLM library (756,096), i.e., to create a Response (index file) for every point in the full CLM. Then, a reduced Predictor (X file) was created for the full CLM using the 15 most important PCs. This reduced the number of columns in the Predictor array (X file) from 1626 to 15 which enabled an SVM training with all elements of the CLM library. This training got 46,633 wrong out of 756,096 which is 6.168 %. This accuracy is not as good as with the training/testing subset, but the accuracy of ~94% is still very good.

Besides reducing dimensionality, the PCs can be investigated in pairs regarding their utility for detecting cancer given an SVM training. A plot of decision equation values vs the scores of PC2 and PC3 has interesting structure as shown in Figure S20. The green rectangle defines plane where the decision-equation equals zero. There is a high degree of structure for both tumor (above zero vertically) and nontumor (below zero vertically) in this plot which uses the two PCs of strongest spectral change. One can study structured regions in Figure S20 by relating these regions to physical morphology in the tissue section imaging. At this point, this is left for future work. The plot in Figure S20 contains 756,096 dots and cannot reveal structure in the high-density regions, so future workers might employ contour strategies and/or slices of such figures to reveal structure in the high-density dot regions. PCA analysis that is informed or supervised by SVM decision equations has potential to provide incisive results that cannot come from PCA alone.

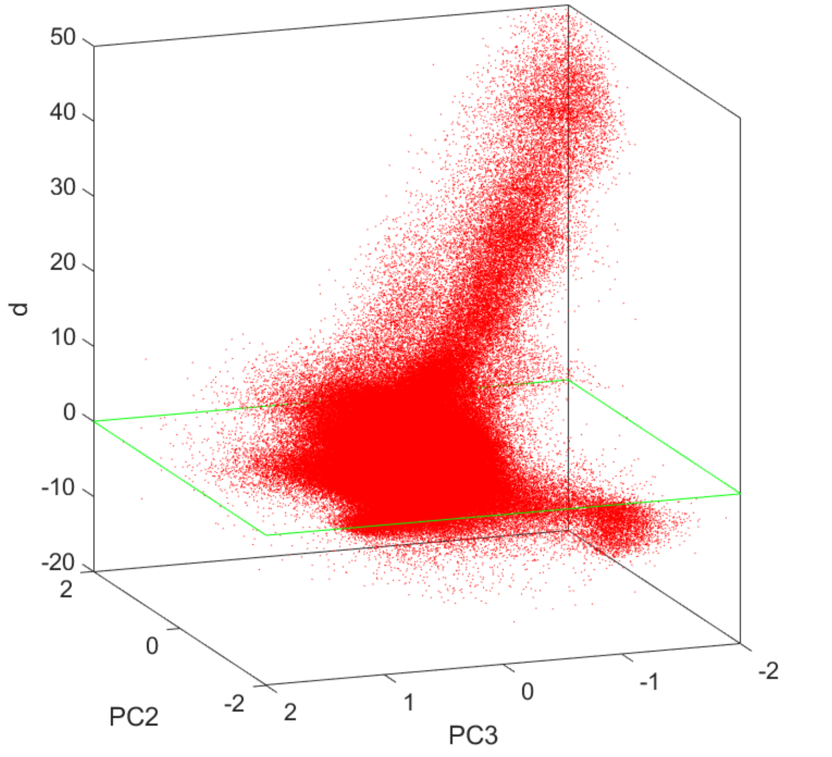

Figure S20. The decision equation values from the 15 PCA analysis of the full CLM library vs the scores of the PC2 and PC3 components. Tumor is above the green rectangle plane, while nontumor is below.

S5.6 *Lymphocytes.* Case 8 was noted in our internal discussions as remarkable because regions rich in lymphocyte cells were readily identified in the H&E images (see Figure 6 in the paper). As already mentioned, the H&E stain reveals purple regions which are regions rich in lymphocyte cells revealing a battle ground against cancer. Note that lymphocyte-rich regions are not yet tumor and are not counted as such by a Pathologist. However, they often become tumor if the patient loses this battle, so a surgical oncologist would want to remove these regions if encountered at the surgical margin. Two boxes were defined with Case 8 for the training and testing of lymphocyte regions as shown in Figure 6b in the paper. A Predictor matrix with 1,136 lymphocyte-rich spectra and 11,657 non-lymphocyte spectra was created giving zero errors in training. The decision equation was used to make an image of Sample Case 8 using purple for lymphocytes as shown in Figure 6c in the paper which matches the H&E stain of Figure 6b in the paper. This lymphocyte decision equation can now be used to search for lymphocytes in other tissue sample cases. The SVM decision equation for lymphocyte-rich regions worked very well in spite of the fact that the lymphocyte-rich IR spectra are very close to tumor spectra. The average IR spectra are shown in Figure S22 using magenta for the lymphocyte-rich spectrum, red for tumor, and green for nontumor.

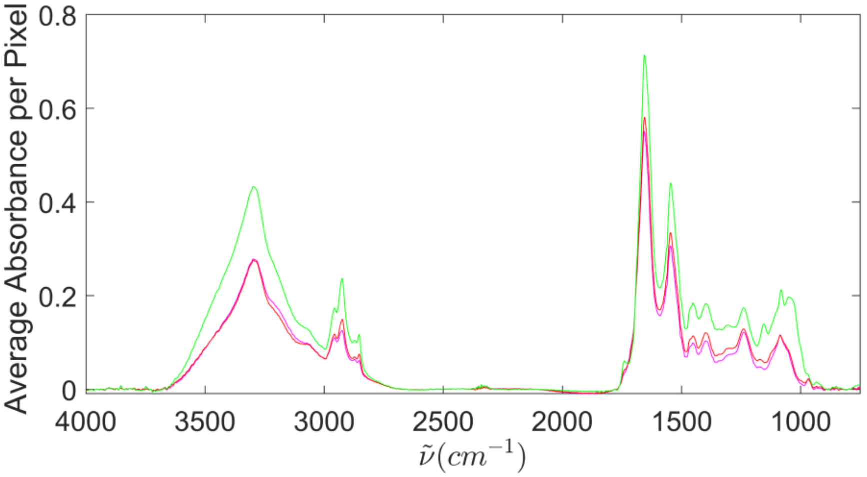

Figure S22. Average IR spectra in Sample Case 8 for the nontumor (green), tumor (red), and the lymphocyte (magenta) regions. The lymphocytes were distinguishable in spite of their similarity to tumor spectra.

References

[1] Bejnordi, B. E.; Litjens, G.; Timofeeva, N.; Otte-Holler, I.; Homeyer, A.; Karssemeijer, N.; van, d. L. J. A. W. M. Stain Specific Standardization of Whole-Slide Histopathological Images. *IEEE Trans. Med. Imaging* **2016**, *35* (2), 404-415.

[2] Riordan, D. P.; Varma, S.; West, R. B.; Brown, P. O. Automated Analysis and Classification of Histological Tissue Features by Multi-dimensional Microscopic Molecular Profiling. *PLoS One* **2015**, *10* (7), e0128975/0128971-e0128975/0128918, 10.1371/journal.pone.0128975. DOI: 10.1371/journal.pone.0128975.

[3] Chekkoury, A.; Khurd, P.; Ni, J.; Bahlmann, C.; Kamen, A.; Patel, A.; Grady, L.; Singh, M.; Groher, M.; Navab, N.; Krupinski, E.; Johnson, J.; Graham, A.; Weinstein, R. Automated Malignancy Detection in Breast Histopathological Images. *Proc. SPIE* **2012**, *8315* (Pt. 1,

Computer-Aided Diagnosis), 831515/831511-831515/831513, 10.1117/12.911643. DOI: 10.1117/12.911643.
[4] Bahlmann, C.; Patel, A.; Johnson, J.; Ni, J.; Chekkoury, A.; Khurd, P.; Kamen, A.; Grady, L.; Krupinski, E.; Graham, A.; Weinstein, R. Automated Detection of Diagnostically Relevant Regions in H&E Stained Digital Pathology Slides. *Proc. SPIE* **2012**, *8315* (Pt. 1, Computer-Aided Diagnosis), 831504/831501-831504/831508, 10.1117/12.912484. DOI: 10.1117/12.912484.
[5] Chapelle, O.; Vapnik, V.; Bousquet, O.; Mukherjee, S. Choosing Multiple Parameters for Support Vector Machines. *Mach. Learn.* **2002**, *46* (1), 131-159.
[6] Le Naour, F.; Bralet, M.-P.; Debois, D.; Sandt, C.; Guettier, C.; Dumas, P.; Brunelle, A.; Laprevote, O. Chemical Imaging on Liver Steatosis using Synchrotron Infrared and ToF-SIMS Microspectroscopies. *PLoS One* **2009**, *4*, No pp. given, 10.1371/journal.pone.0007408. DOI: 10.1371/journal.pone.0007408.
[7] Debois, D.; Bralet, M.-P.; Le Naour, F.; Brunelle, A.; Laprevote, O. In Situ Lipidomic Analysis of Nonalcoholic Fatty Liver by Cluster TOF-SIMS Imaging. *Anal. Chem. (Washington, DC, U. S.)* **2009**, *81* (8), 2823-2831, 10.1021/ac900045m. DOI: 10.1021/ac900045m.
[8] Abdel-Misih, S. R. Z.; Schmidt, C. R.; Bloomston, P. M. Update and Review of the Multidisciplinary Management of Stage IV Colorectal Cancer with Liver Metastases. *World J. Surg. Oncol.* **2009**, *7*, 72.
[9] Fernandez, D. C.; Bhargava, R.; Hewitt, S. M.; Levin, I. W. Infrared Spectroscopic Imaging for Histopathologic Recognition. *Nature Biotechnol.* **2005**, *23* (4), 469-474, Article. DOI: 10.1038/nbt1080.
[10] Chen, Z.; Butke, R.; Miller, B.; Hitchcock, C. L.; Allen, H. C.; Povoski, S. P.; Martin, E. W.; Coe, J. V. Infrared Metrics for Fixation-Free Liver Tumor Detection. *J. Phys. Chem. B* **2013**, *117* (41), 12442-12450. DOI: 10.1021/jp4073087.
[11] Ayyıldız, S. N.; Ayyıldız, A. Cyanoacrylic Tissue Glues: Biochemical Properties and Their Usage in Urology. *Turkish Journal of Urology* **2017**, *43* (1), 14.
[12] Zoccali, C.; Covello, R.; Di Francesco, A.; Zoccali, G. A Cyanoacrylate and Silastic Patch to Reduce the Risk of Opening of the Tumor. *Eur. J. Surg. Oncol.* **2013**, *39* (1), 44-45.
